# Supplementary material for: Applying a Chemogeographic Strategy for Natural Product Discovery from the Marine Cyanobacterium Moorena bouillonii
Source: Mar Drugs. 2020 Oct 14;18(10):515. doi: 10.3390/md18100515 (PMC7602127; doi:10.3390/md18100515)
Supplement: Supplementary file 1 [file marinedrugs-18-00515-s001.pdf]

marine drugs

MDPI

Supporting Information

Applying a Chemogeographic Strategy for Natural Product Discovery from the Marine Cyanobacterium *Moorena bouillonii*

Christopher A. Leber [1], C. Benjamin Naman[1,2], Lena Keller[1,3], Jehad Almaliti[1,4], Eduardo J. E. Caro-Diaz[1,5], Evgenia Glukhov[1], Valsamma Joseph[1,6], T. P. Sajeevan[1,6], Andres Joshua Reyes[7], Jason S. Biggs[7], Te Li[2], Ye Yuan[2], Shan He[2], Xiaojun Yan[2], and William H. Gerwick [1,8,*]

[1] Center for Marine Biotechnology and Biomedicine, Scripps Institution of Oceanography, University of California San Diego, La Jolla, California 92093, United States; cleber@ucsd.edu (C.A.L.); bnaman@nbu.edu.cn (C.B.N.); le.keller85@gmail.com (L.K.); jalmaliti@ucsd.edu (J.A.); eduardo.caro1@upr.edu (E.J.E.C.D.); eglukhov@ucsd.edu (E.G.); valsamma@cusat.ac.in (V.J.); sajeev@cusat.ac.in (T.P.S.)

[2] Li Dak Sum Yip Yio Chin Kenneth Li Marine Biopharmaceutical Research Center, Department of Marine Pharmacy, College of Food and Pharmaceutical Sciences, Ningbo University, Ningbo, China; telinbu@163.com (T.L.); 23yuanye@163.com (Y.Y.); heshan@nbu.edu.cn (S.H.); yanxiaojun@nbu.edu.cn (X.Y.)

[3] Department Microbial Natural Products, Helmholtz-Institute for Pharmaceutical Research Saarland (HIPS), Helmholtz Centre for Infection Research (HZI), Campus E8.1, 66123 Saarbrücken, Germany

[4] School of Pharmacy, The University of Jordan, Amman, Jordan

[5] Department of Pharmaceutical Sciences, School of Pharmacy, University of Puerto Rico – Medical Sciences Campus, San Juan, Puerto Rico 00921

[6] National Centre for Aquatic Animal Health, Cochin University of Science and Technology, Kochi, Kerala 682016, India

[7] University of Guam Marine Laboratory, Mangilao, Guam 96923; reyes.andresjoshua@gmail.com (A.J.R.); biggs.js@gmail.com (J.S.B.)

[8] Skaggs School of Pharmacy and Pharmaceutical Sciences, University of California San Diego, La Jolla, California 92093, United States

* Correspondence: wgerwick@health.ucsd.edu (W.H.G.)

Received: XX; Accepted: XX; Published: XX

Table of Contents

Figure S1. Molecular network of *M. bouillonii* crude extracts S3
Figure S2. Predicted ^{13}C shifts for candidate structure **1a** S4
Figure S3. Predicted ^{13}C shifts for candidate structure **1b** S4
Figure S4. Compound **1** derived 2-methyoctanoic acid compared to standards S5
Figure S5. Compound **1** derived lysine compared to standards S6
Figure S6. Doscadenamide A (**1**) consensus MS^2 spectrum S7
Figure S7. Molecular network cluster of compound **1** and analogs, highlighting *m*/*z* 168 frag. peak S8
Figure S8. Structure of compound **1** with structure proposals for analogs (**2-10**) S9
Figure S9. Doscadenamide B (**2**) consensus MS^2 spectrum S10
Figure S10. Doscadenamide B (**2**) proposed fragmentation S11
Figure S11. Doscadenamide C (**3**) consensus MS^2 spectrum S10
Figure S12. Doscadenamide C (**3**) proposed fragmentation S13
Figure S13. Doscadenamide D (**4**) consensus MS^2 spectrum S14
Figure S14. Doscadenamide D (**4**) proposed fragmentation S15

Figure S15. Doscadenamide E (**5**) consensus MS^2 spectrum S16
Figure S16. Doscadenamide E (**5**) proposed fragmentation S17
Figure S17. Doscadenamide F (**6**) consensus MS_2 spectrum S18
Figure S18. Doscadenamide F (**6**) proposed fragmentation S19
Figure S19. Doscadenamide G (**7**) consensus MS^2 spectrum S20
Figure S20. Doscadenamide G (**7**) proposed fragmentation S21
Figure S21. Doscadenamide H (**8**) consensus MS^2 spectrum S22
Figure S22. Doscadenamide H (**8**) proposed fragmentation S23
Figure S23. Doscadenamide I (**9**) consensus MS^2 spectrum S24
Figure S24. Doscadenamide I (**9**) proposed fragmentation S25
Figure S25. Doscadenamide J (**10**) consensus MS^2 spectrum S26
Figure S26. Doscadenamide J (**10**) proposed fragmentation S27
Figure S27. Representative structures from compound families similar to the doscadenamides S28
Figure S28. Results of compound **1** in Griess assay – biological replicate 1 S29
Figure S29. Results of compound **1** in Griess assay – biological replicate 2 S29
Figure S30. Results of compound **1** in Griess assay – biological replicate 3 S30
Figure S31. UV/Vis absorbance spectrum (200-400 nm) for compound **1** S30
Figure S32. IR spectrum for compound **1** S31
Figure S33. ^1H NMR spectrum for compound **1** S31
Figure S34. ^{13}C NMR spectrum for compound **1** S32
Figure S35. ^1H-^1H COSY spectrum for compound **1** S33
Figure S36. ^1H-^{13}C HSQC spectrum for compound **1** S33
Figure S37. ^1H-^{13}C HMBC spectrum for compound **1** S34
Figure S38. ^1H-^{13}C HSQC-TOCSY spectrum for compound **1** S34
Figure S39. ^1H-^{13}C Long-range HSQMBC spectrum for compound **1** S35

Table S1. Known compounds isolated from *M. bouillonii* S36
Table S2. Average relative abundances and feature selection scores for top 10 Saipan MS^1 features S37
Table S3. Putative identifications for top 30 MS^1 features in the *M. bouillonii* crude extract dataset S38
Table S4. *In silico* antibiotic screening results for the doscadenamides and tetramic acids S38
Table S5. *M. bouillonii* crude extract sample metadata S39
Table S6. ORCA parameter set for MS^1 feature dendrogram S39
Table S7. ORCA parameter set for GNPS MS^2 feature presence/absence dendrogram S40
Table S8. ORCA parameter set for MS^1 feature selection S40
Table S9. GNPS parameter set for *M. bouillonii* crude extract molecular network S41
Table S10. GNPS parameter set for *M. bouillonii* crude extract MS^2 feature bucket table S41
Table S11. GNPS parameter set for Saipan and Guam crude extracts and fractions molecular network S42
Table S12. VLC fractionation solvent systems S42
Table S13. ^1H and ^{13}C NMR Data for doscadenamide A (**1**) in $CDCl_3$ S43

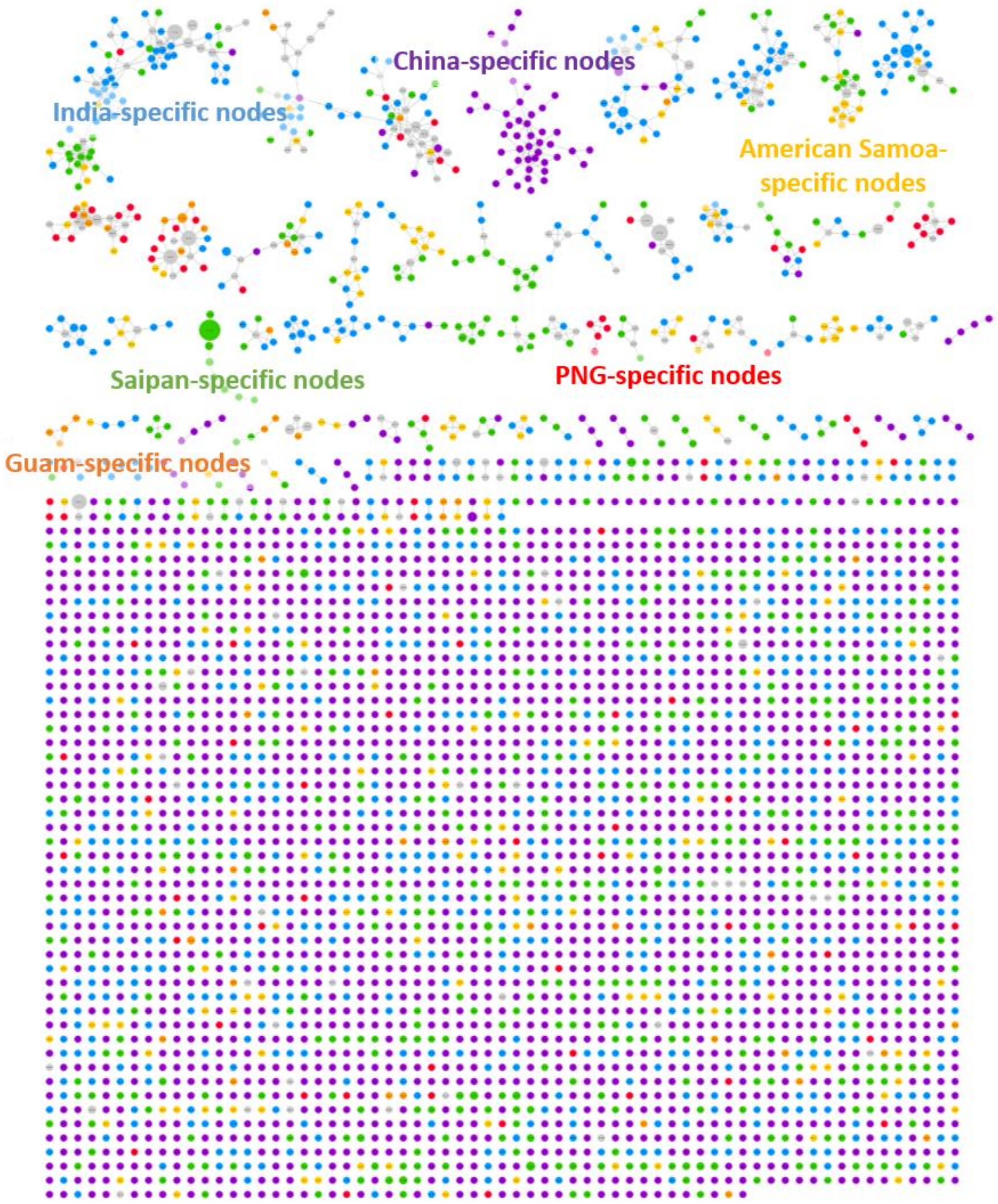

Figure S1. Molecular network of M. bouillonii crude extracts

GNPS classical molecular network of *M. bouillonii* crude extracts showing clusters of regionally specific nodes. Grey nodes represent MS^2 features that are present in samples from more than one geographically region. Nodes are scaled to summed precursor intensity. Red: Papua New Guinea, Orange: Guam, Gold: American Samoa, Green: Saipan, Blue: Kavaratti (Lakshadweep Islands, India), Purple: Xisha (Paracel) Islands in the South China Sea. See Table S9 for network parameters.

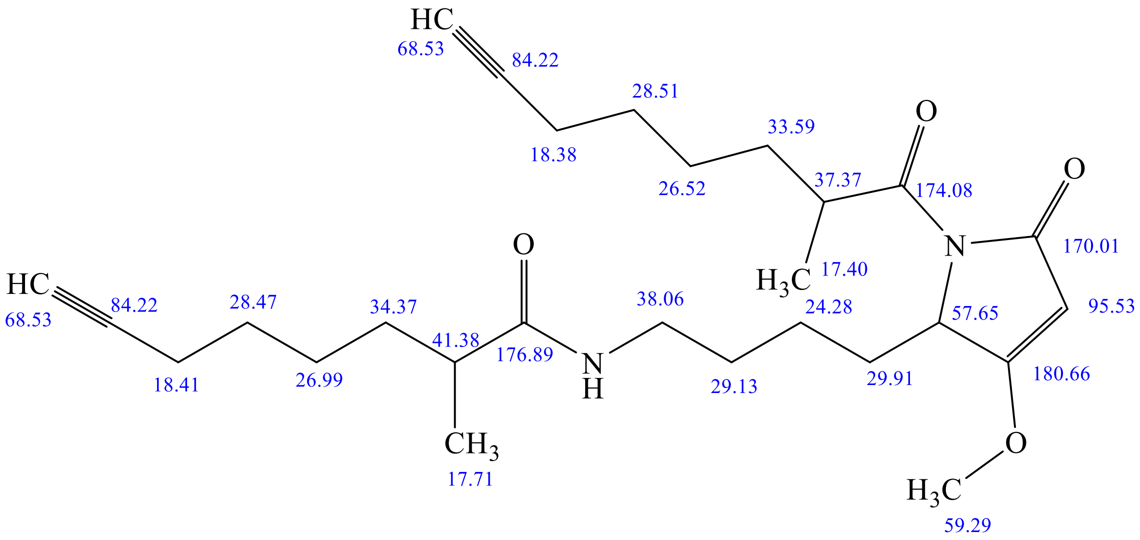

Figure S2. Predicted ^{13}C shifts for candidate structure **1a**

^{13}C NMR shifts were calculated using ACD/Labs 2019.2.1 (ACD/C+H Predictors and DB 2019.2.1) (https://www.acdlabs.com/index.php).

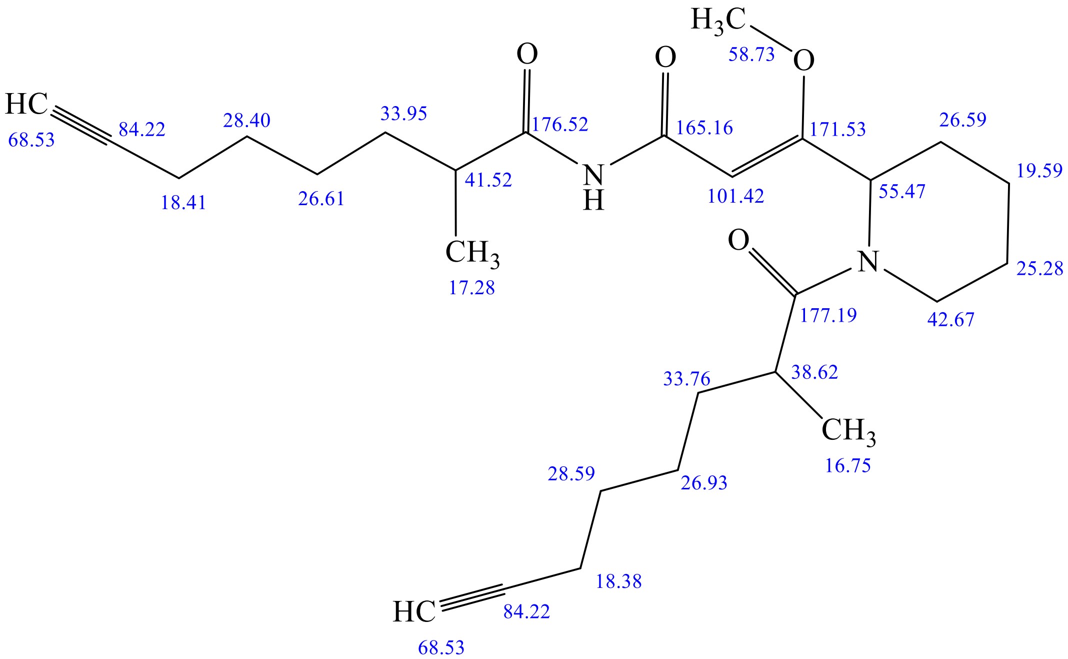

Figure S3. Predicted ^{13}C shifts for candidate structure **1b**

^{13}C NMR shifts were calculated using ACD/Labs 2019.2.1 (ACD/C+H Predictors and DB 2019.2.1) (https://www.acdlabs.com/index.php).

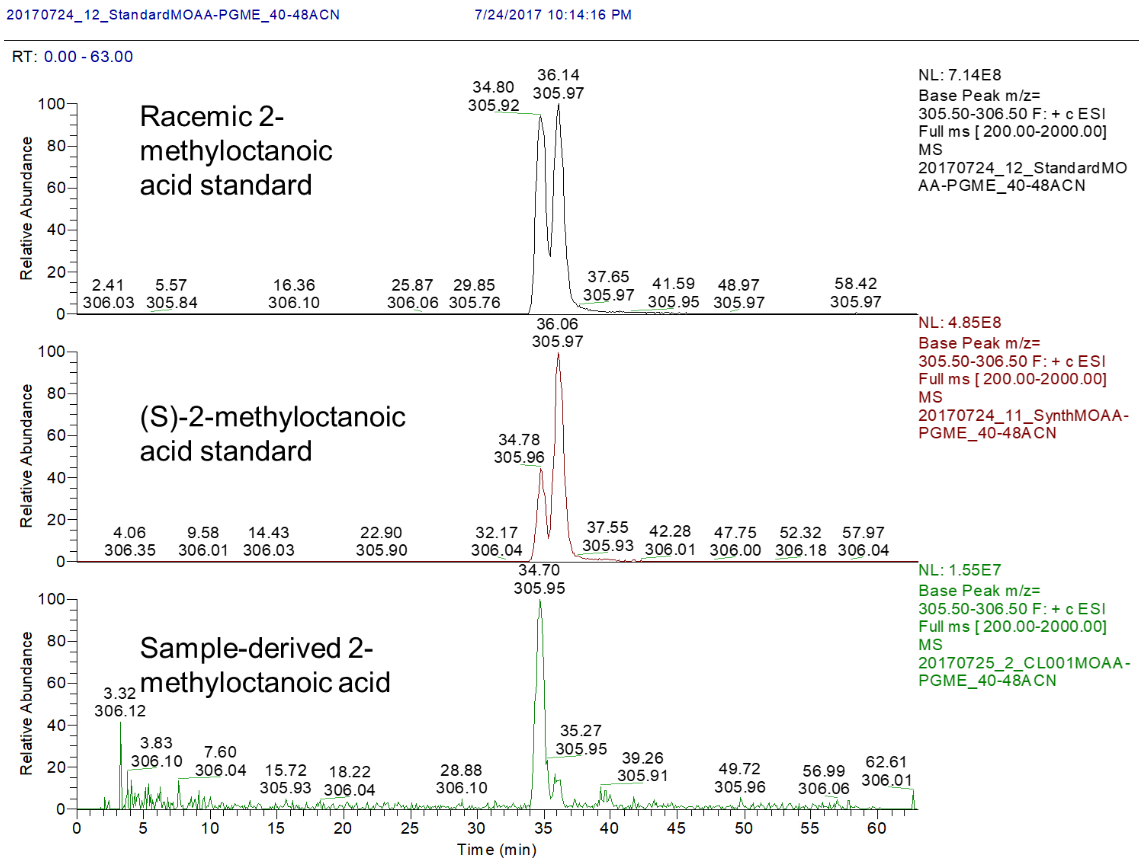

Figure S4. Compound **1** derived 2-methyoctanoic acid compared to standards

LC-MS TIC traces comparing (*S*)-(+)-2-phenylglycine methyl ester derivatized racemic 2-methyloctanoic acid and (*S*)-2-methyloctanoic acid standards to sample-derived 2-methyl octanoic acid, indicating the sample-derived 2-methyl octanoic acid to be of the *R* configuration.

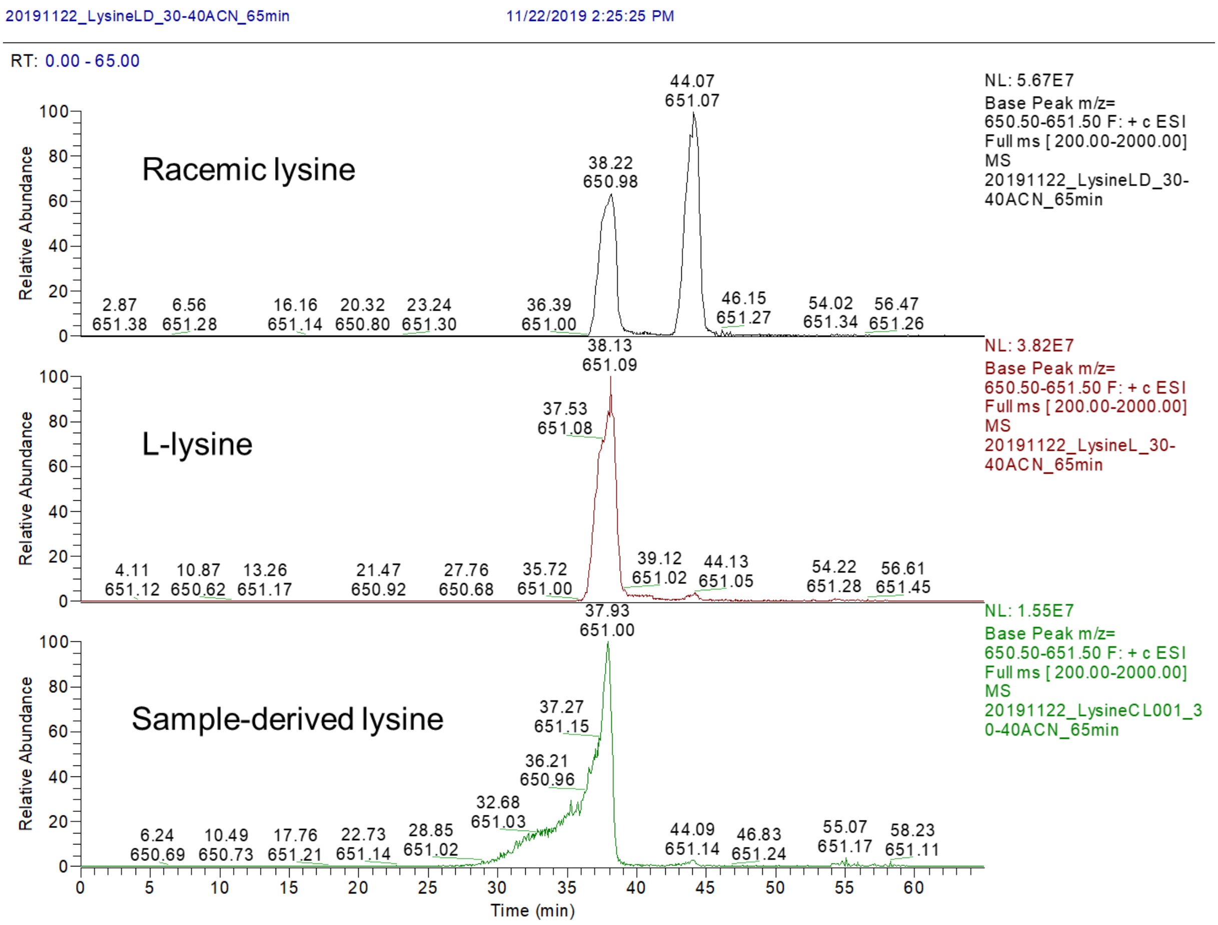

Figure S5. Compound **1** derived lysine compared to standards
LC-MS TIC traces comparing L-FDAA derivatized racemic lysine and L-lysine standards to sample-derived lysine, indicating the sample-derived lysine to be of the *S* configuration (L-lysine).

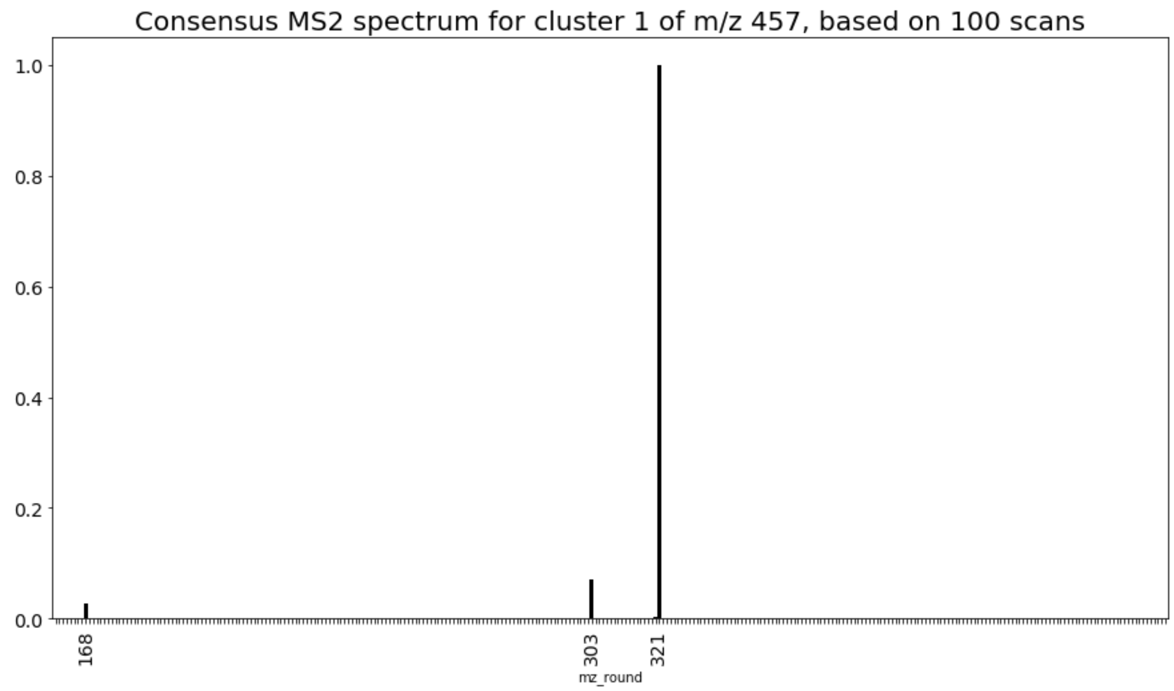

Figure S6. Doscadenamide A (**1**) consensus MS^2 spectrum

Consensus MS^2 spectrum representing a cluster of 100 scans for precursor mass *m*/*z* 457 and displaying the fragmentation spectrum for doscadenamide A (**1**).

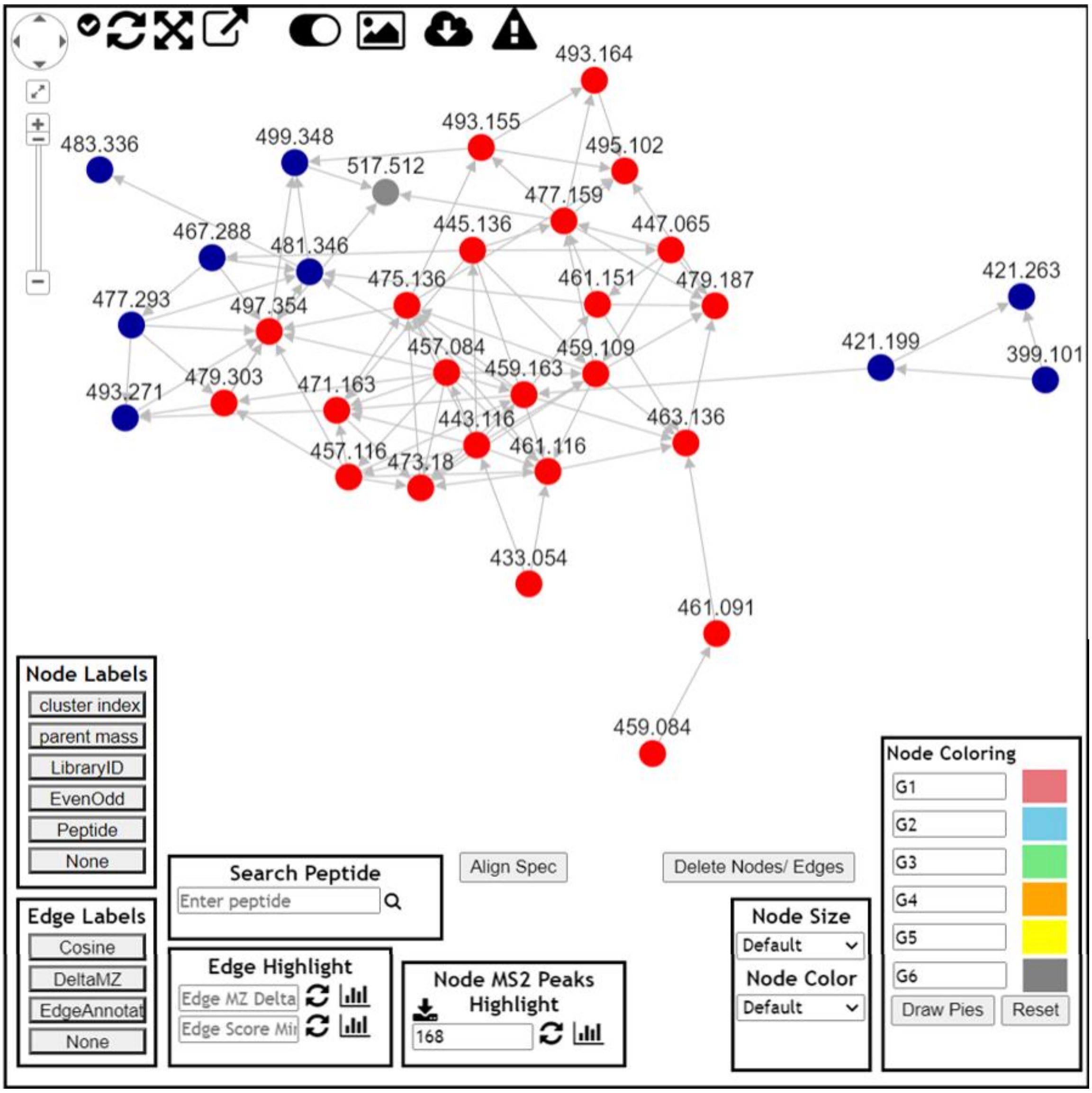

Figure S7. Molecular network cluster of compound **1** and analogs, highlighting *m/z* 168 frag. peak
A cluster of 33 MS^2 spectral nodes, including compound **1** (*m/z* 457.084), as visualized in the GNPS in browser network visualizer. This cluster is a part of a GNPS classical molecular network generated with crude extracts and fractions from two *M. bouillonii* samples: one from Saipan and one from Guam. All nodes colored red (23 out of 33) possess a fragment peak at *m/z* 168, suggesting that the structures they represent include a heterocyclic core identical to compound **1**.

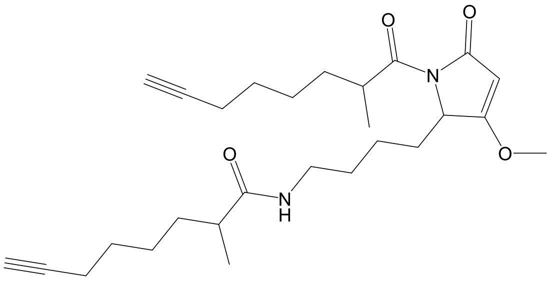

1 doscadenamide A

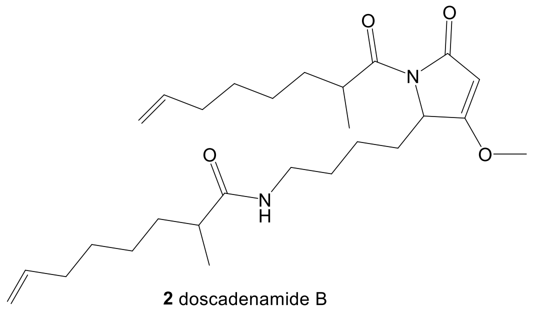

2 doscadenamide B

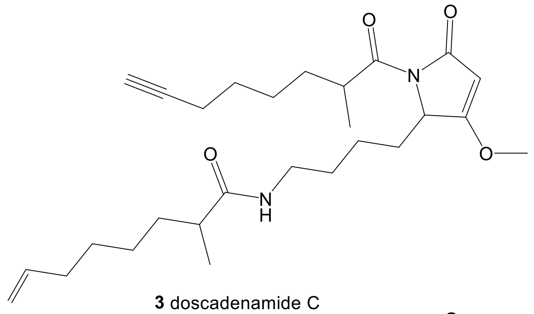

3 doscadenamide C

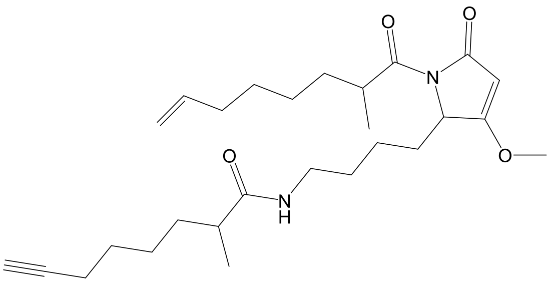

4 doscadenamide D

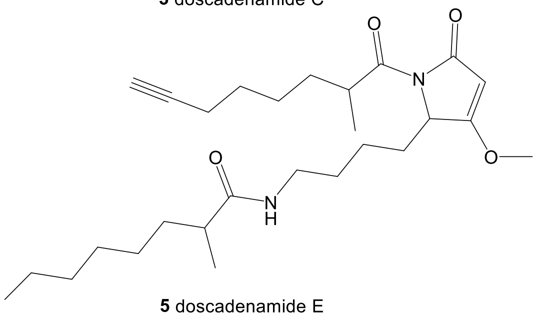

5 doscadenamide E

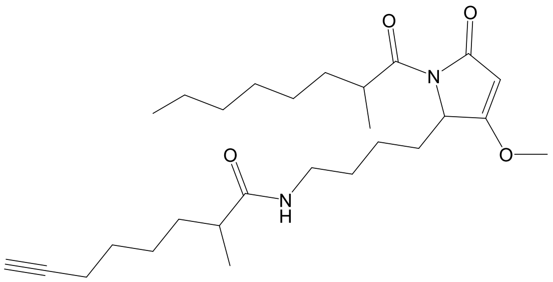

6 doscadenamide F

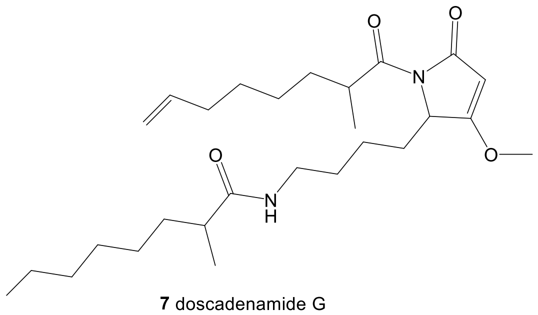

7 doscadenamide G

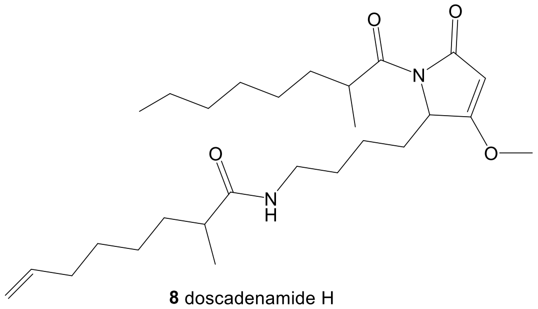

8 doscadenamide H

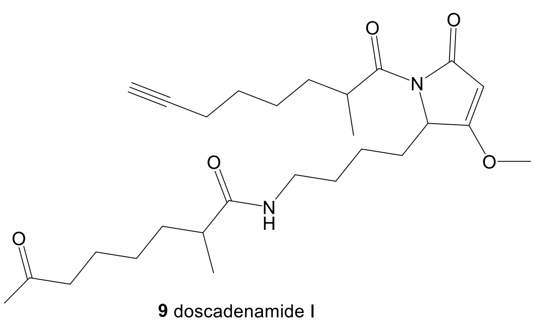

9 doscadenamide I

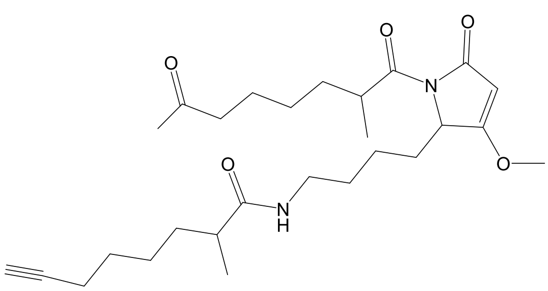

10 doscadenamide J

Figure S8. Structure of compound **1** with structure proposals for analogs (**2-10**)
The doscadenamides: compound **1**, along with analogs whose proposed structures were annotated via informative patterns in the MS² fragmentation data (See Figures S9-S26).

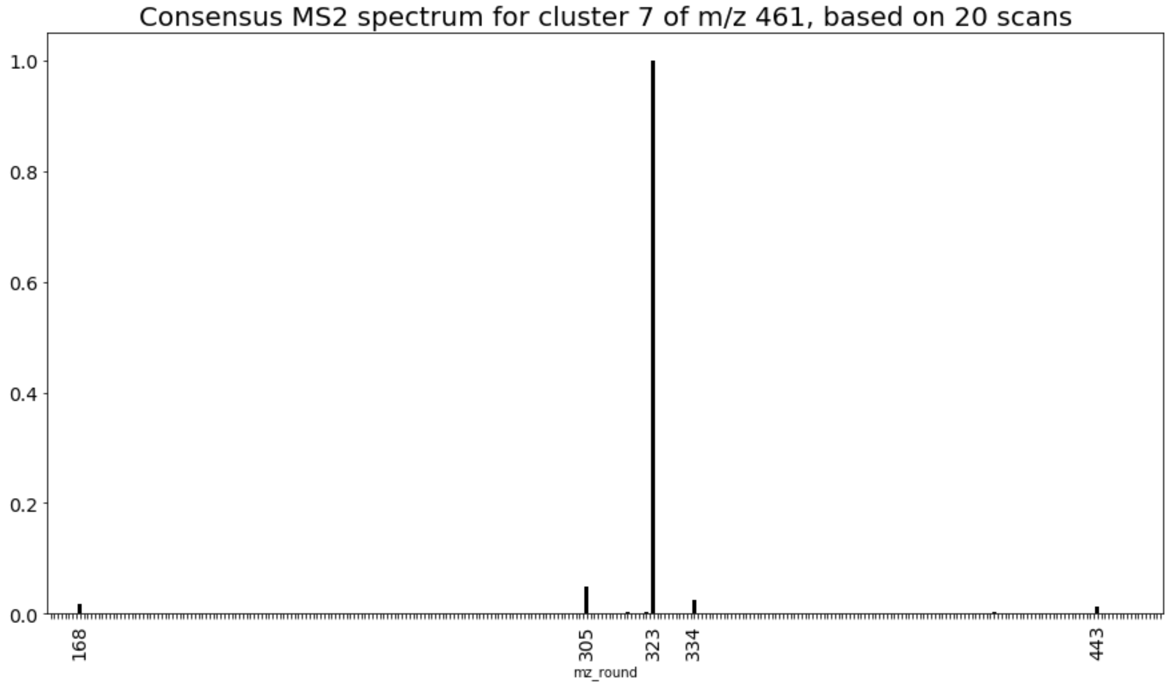

Figure S9. Doscadenamide B (**2**) consensus MS^2 spectrum
Consensus MS^2 spectrum representing a cluster of 20 scans for precursor mass *m/z* 461, representing the fragmentation spectrum of the proposed analog doscadenamide B (**2**).

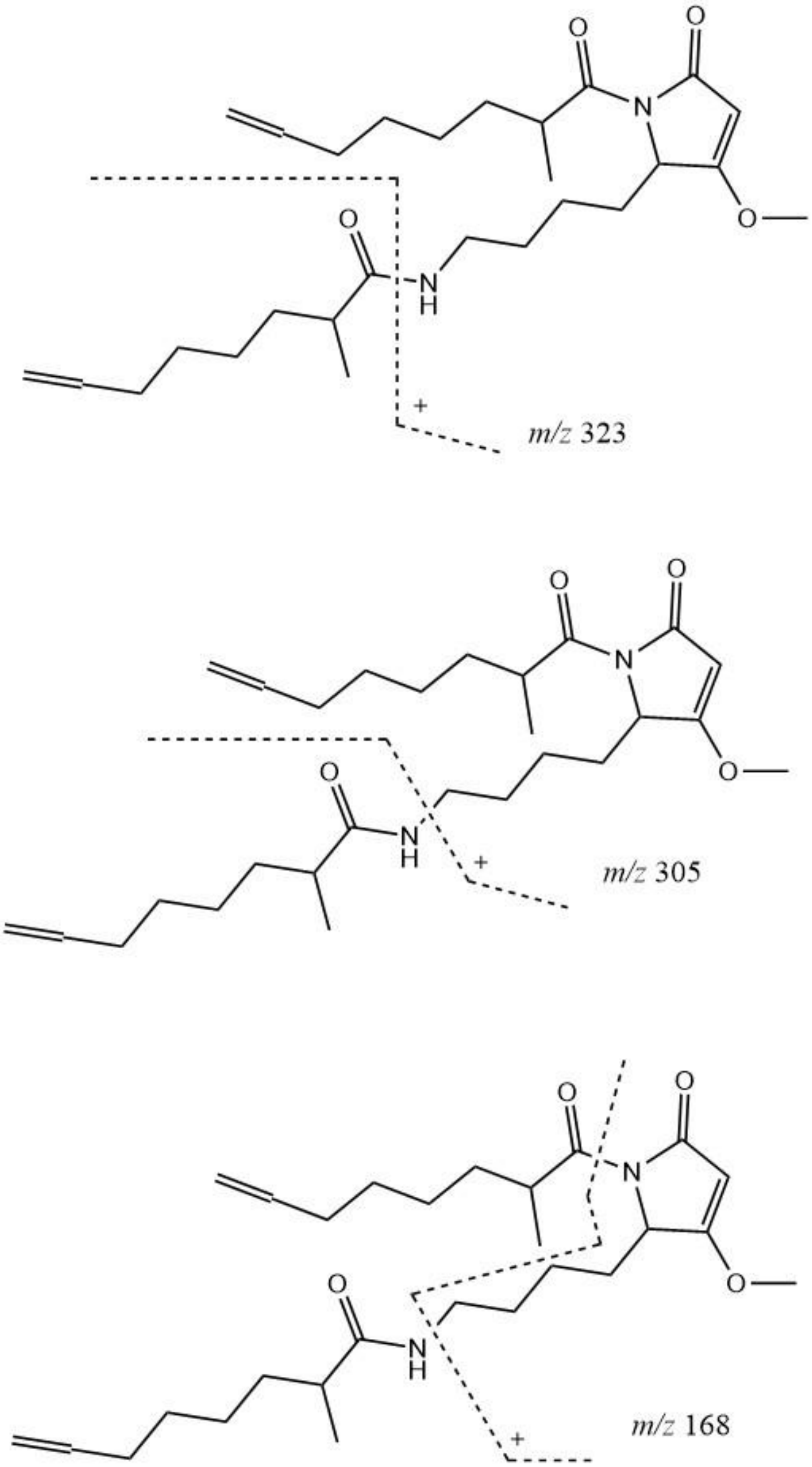

Figure S10. Doscadenamide B (**2**) proposed fragmentation

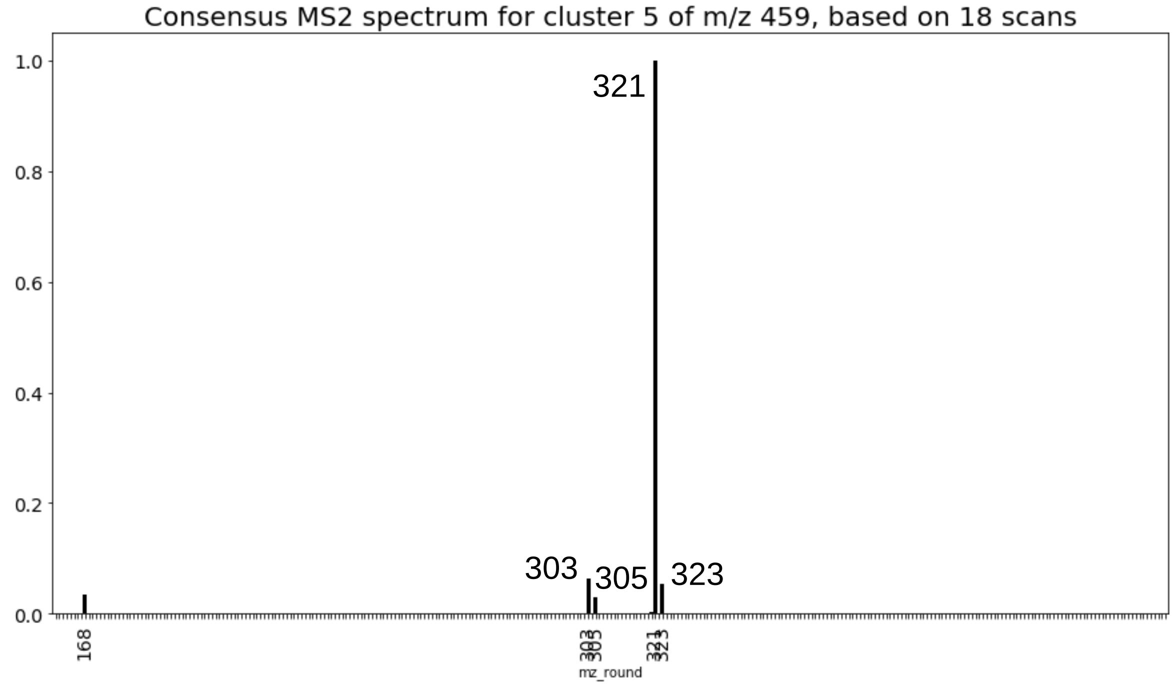

Figure S11. Doscadenamide C (**3**) consensus MS² spectrum
Consensus MS² spectrum representing a cluster of 18 scans for precursor mass *m*/*z* 459, representing the fragmentation spectrum of the proposed analog doscadenamide C (**3**).

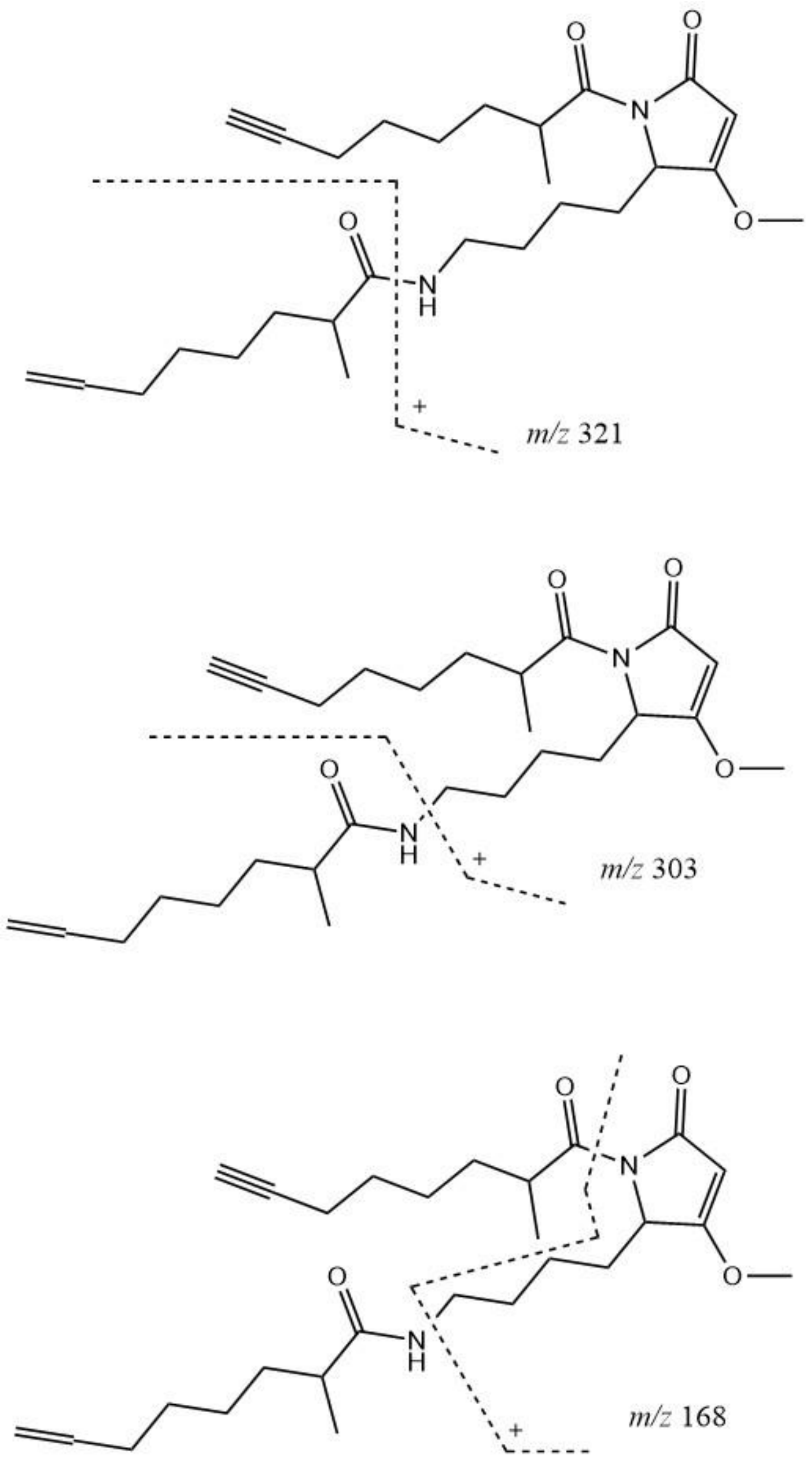

Figure S12. Doscadenamide C (**3**) proposed fragmentation

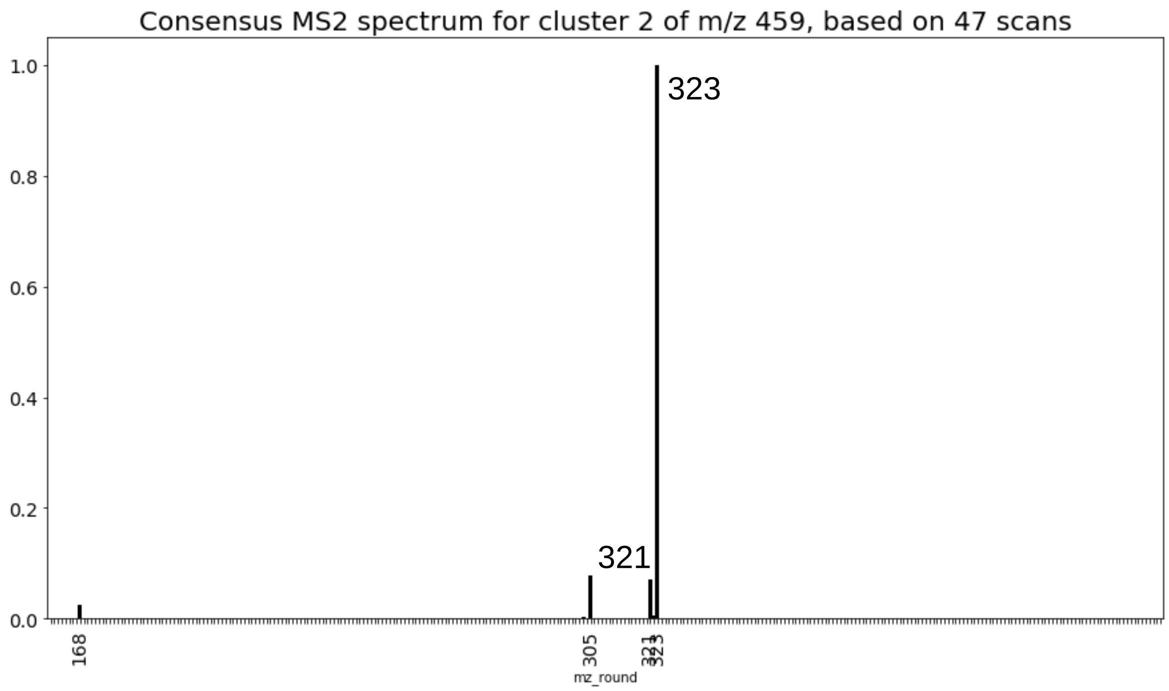

Figure S13. Doscadenamide D (**4**) consensus MS^2 spectrum
Consensus MS^2 spectrum representing a cluster of 47 scans for precursor mass *m*/*z* 459, representing the fragmentation spectrum of the proposed analog doscadenamide D (**4**).

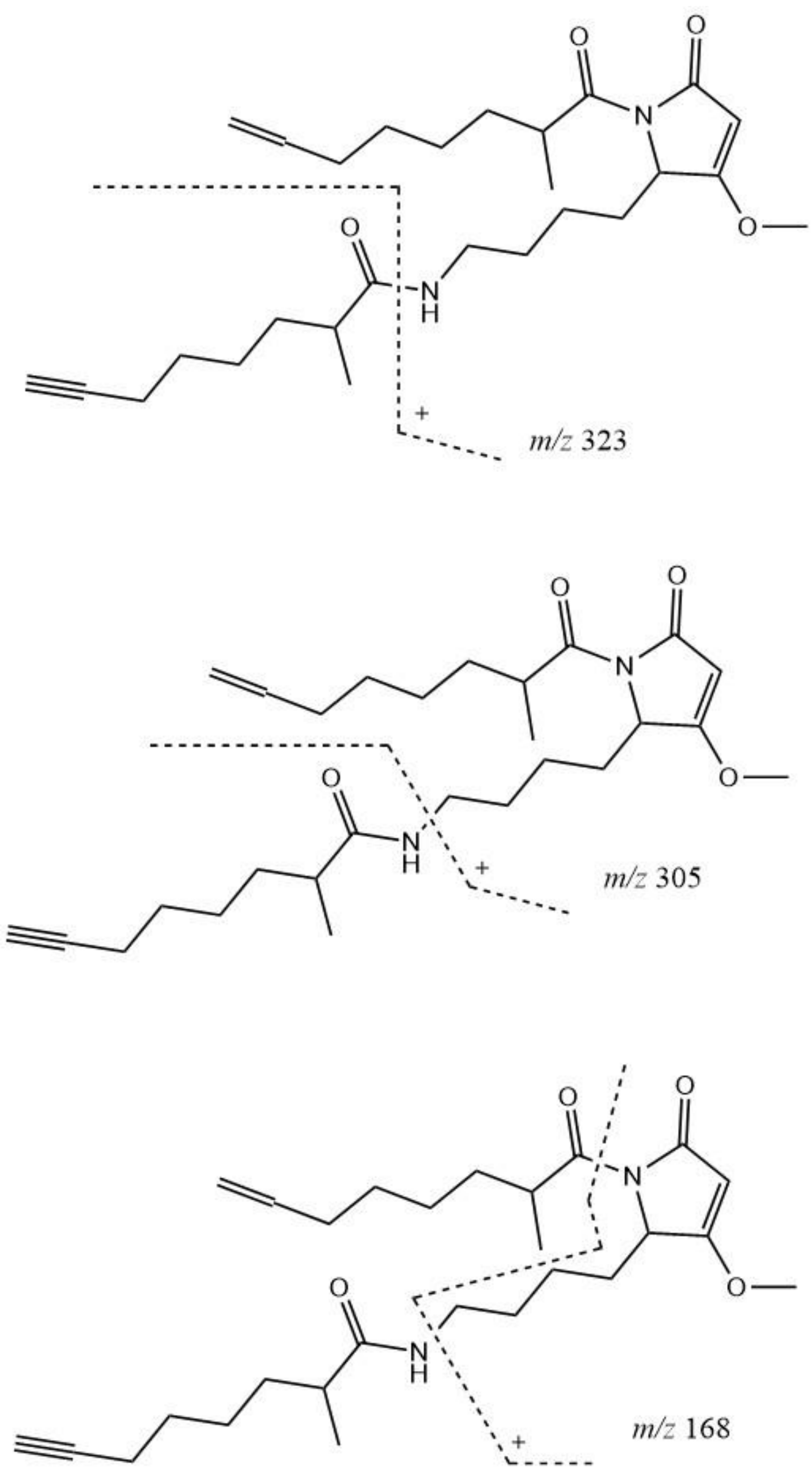

Figure S14. Doscadenamide D (**4**) proposed fragmentation

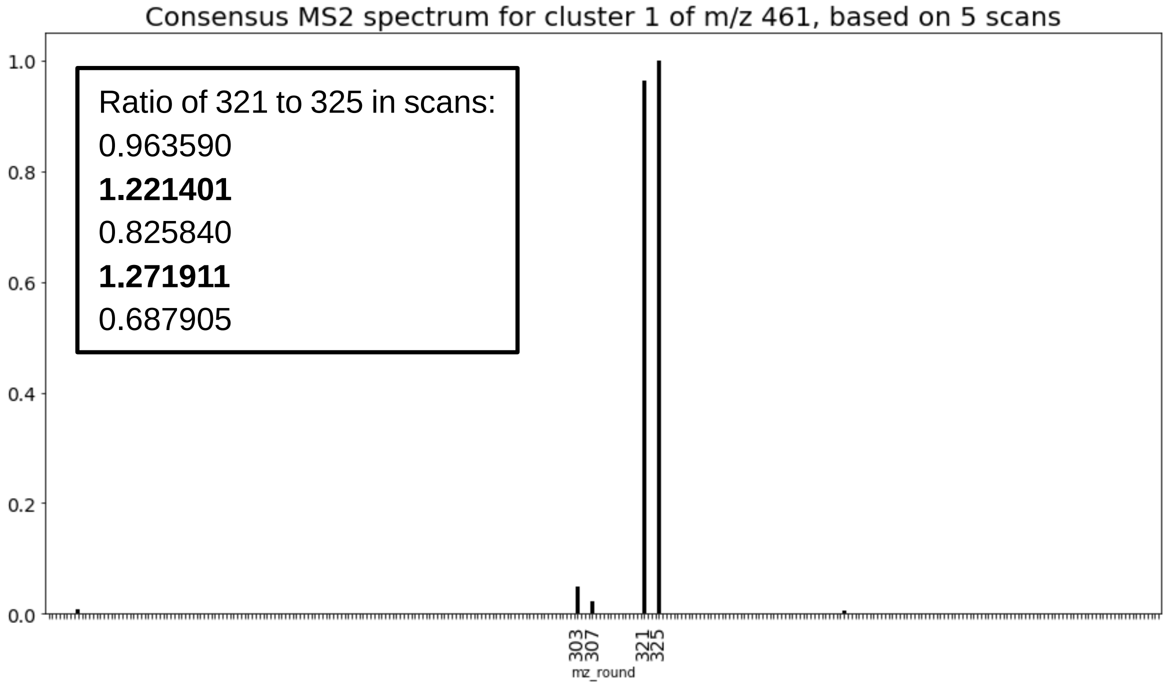

Figure S15. Doscadenamide E (**5**) consensus MS^2 spectrum
Consensus MS^2 spectrum representing a cluster of 5 scans for precursor mass *m/z* 461, representing the fragmentation spectrum of the proposed analog doscadenamide E (**5**). The expected fragmentation spectrum would have a fragment peak at *m/z* 321 that is more intense than the fragment peak at *m/z* 325, indicating the apparent propensity for side chains acylated to the terminus of the lysine side chain to fragment. The inlay in the top right hand corner reports the ratio of the *m/z* 321 peak relative intensity to *m/z* 325 peak relative intensity for the 5 scans represented in the consensus. These ratios reveal that in 2 scans, the fragment peak at *m/z* 321 is indeed more intense than the fragment peak at *m/z* 325.

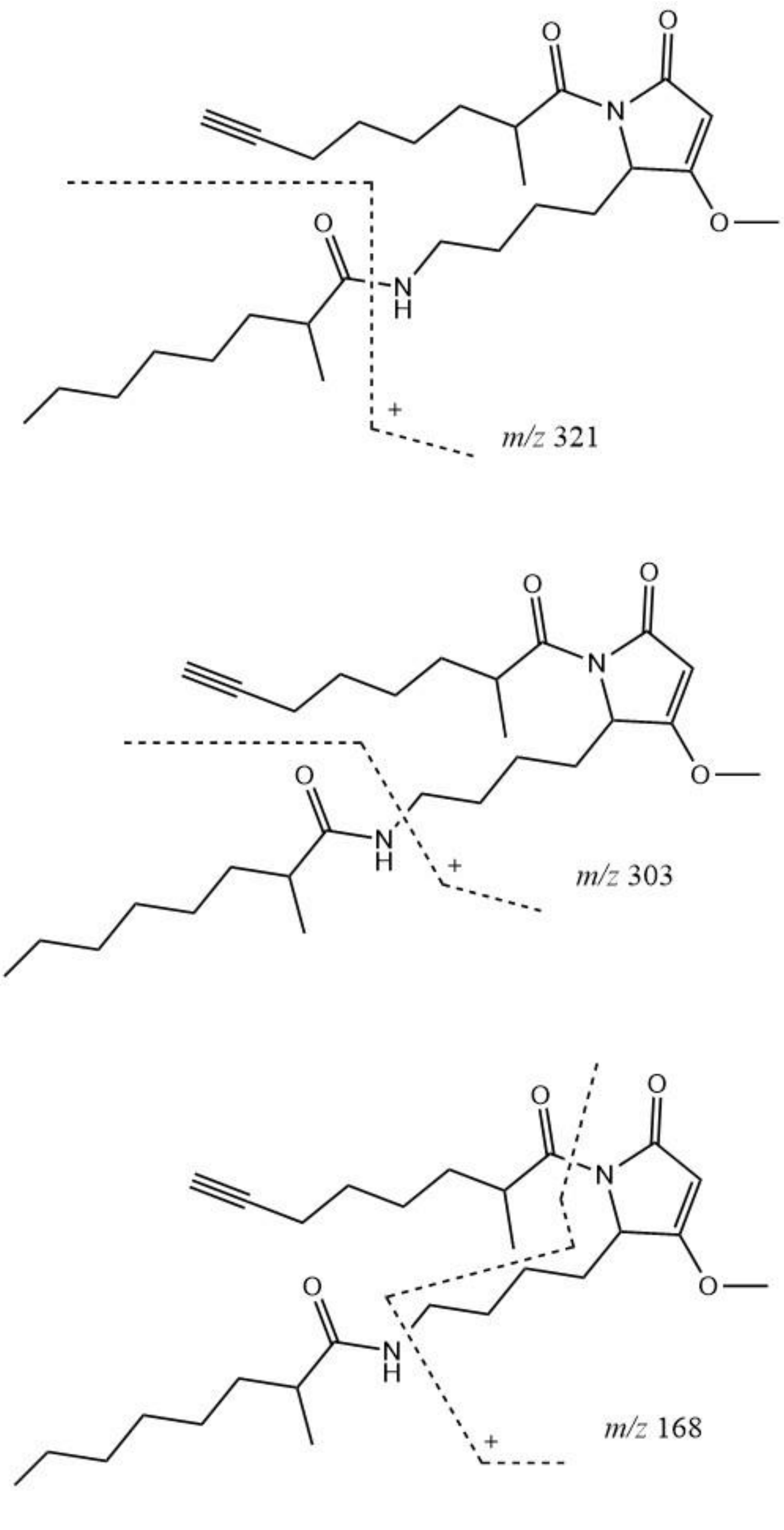

Figure S16. Doscadenamide E (**5**) proposed fragmentation

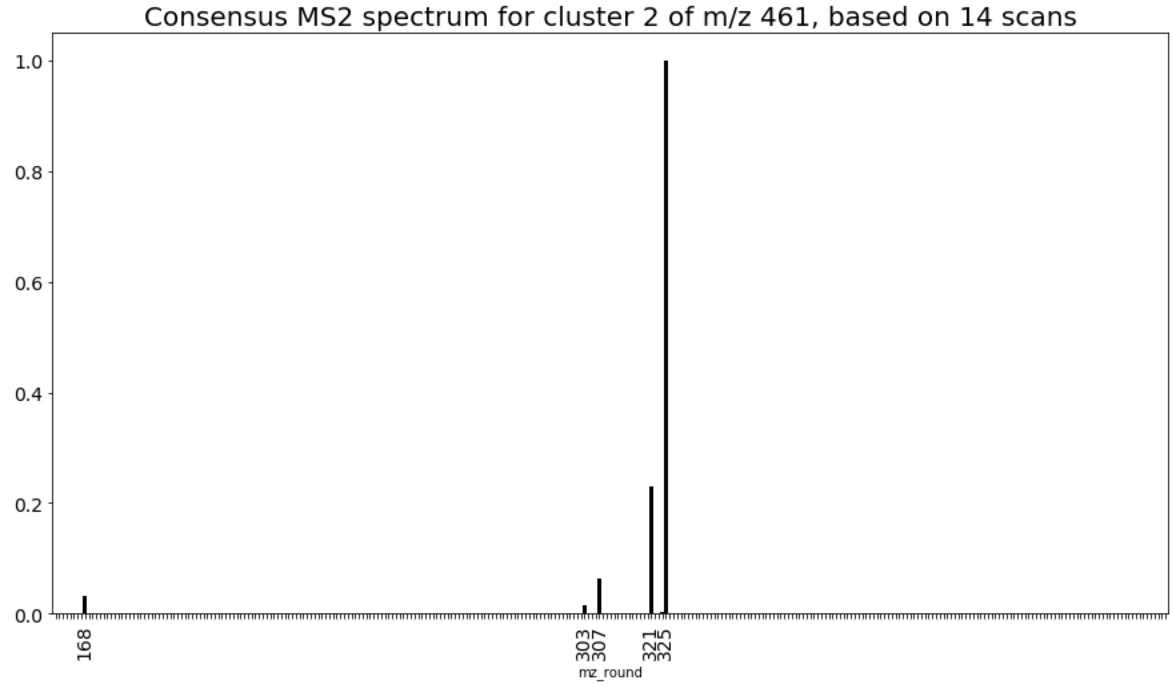

Figure S17. Doscadenamide F (**6**) consensus MS_2 spectrum
Consensus MS^2 spectrum representing a cluster of 14 scans for precursor mass *m*/*z* 461, representing the fragmentation spectrum of the proposed analog doscadenamide F (**6**).

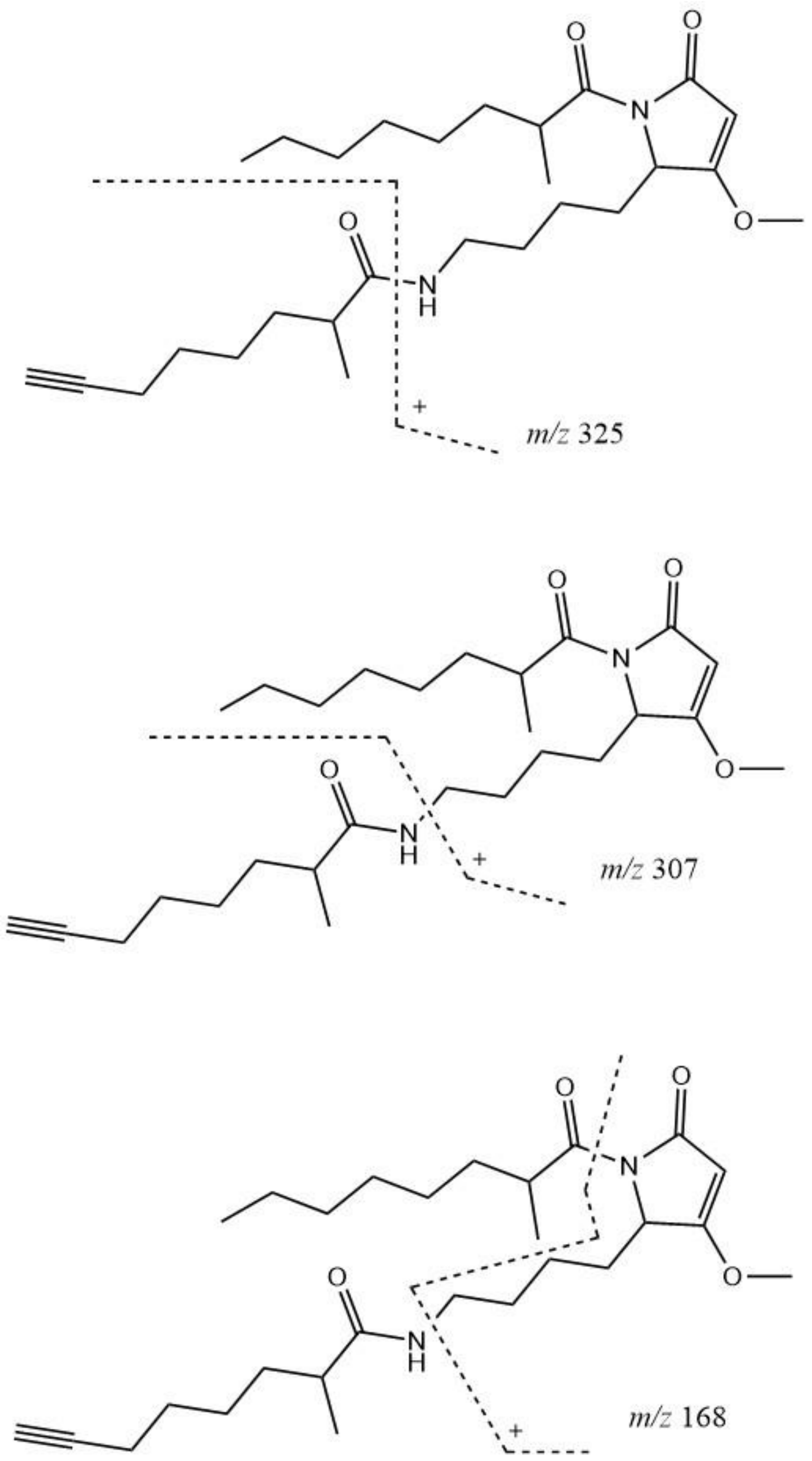

Figure S18. Doscadenamide F (**6**) proposed fragmentation

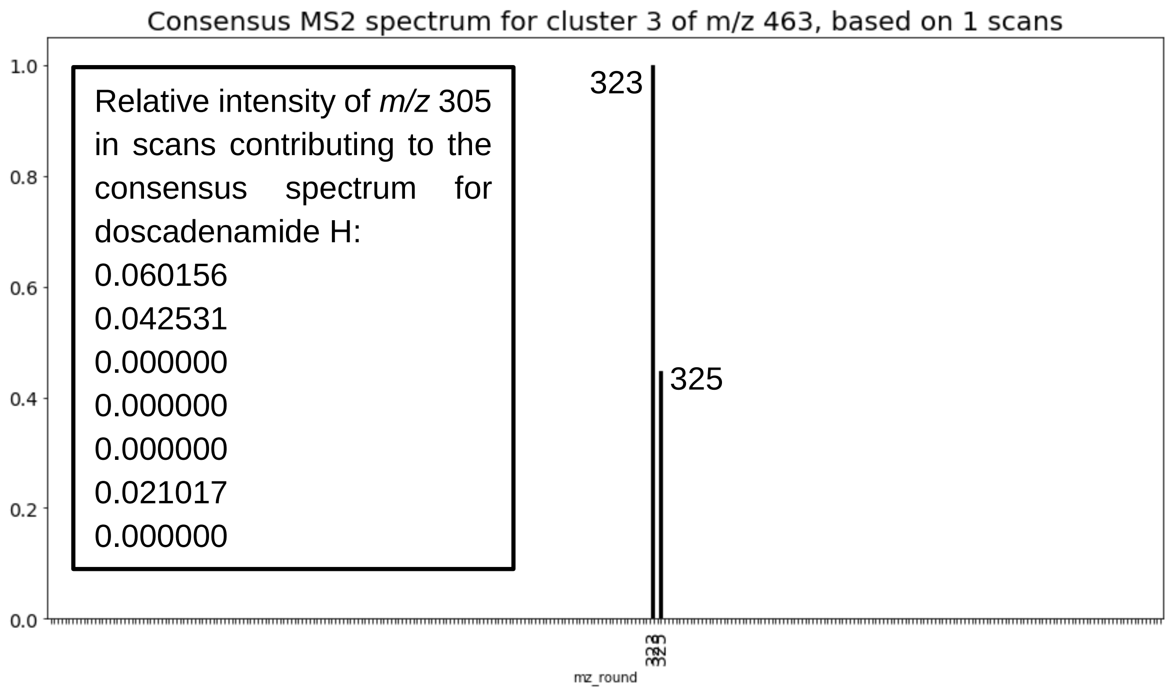

Figure S19. Doscadenamide G (**7**) consensus MS^2 spectrum
MS^2 spectrum captured in one scan for precursor mass *m*/*z* 463, representing the partial fragmentation spectrum of the proposed analog doscadenamide G (**7**). The expected *m*/*z* 305 and *m*/*z* 168 peaks were detected at too low of intensity to appear in this output. However, the *m*/*z* 305 peak is detected in several of the scans that make up the consensus spectrum representing doscadenamide H (Figure S20) – the relative intensity of these *m*/*z* 305 fragment peaks are displayed in the above figure inlay. Detection of this m/z 305 peak is important because doscadenamides G and H coelute and such a fragment peak would not be produced by doscadenamide H. Therefore, this lends further support to the structural proposal for doscadenamide G.

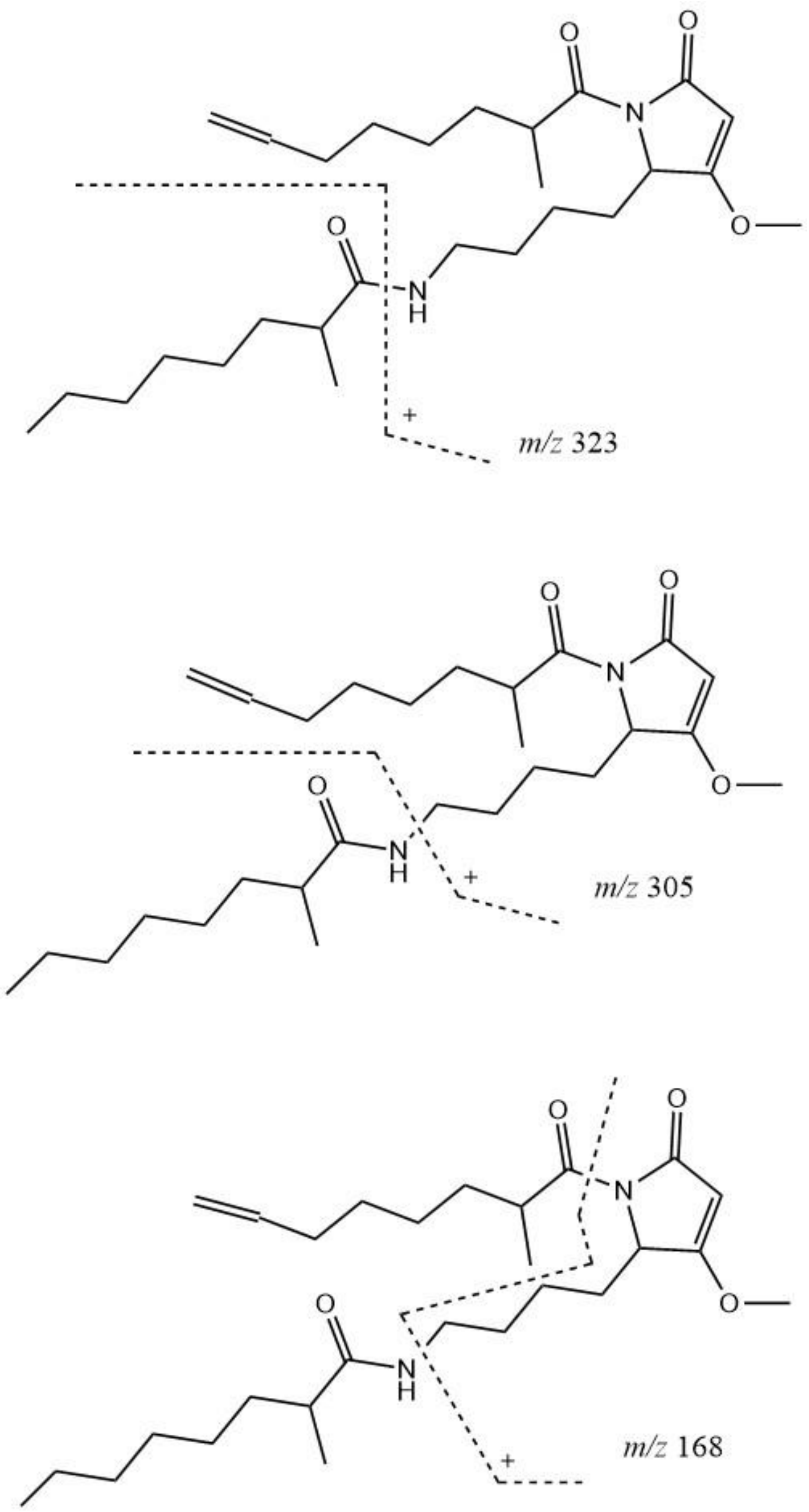

Figure S20. Doscadenamide G (**7**) proposed fragmentation

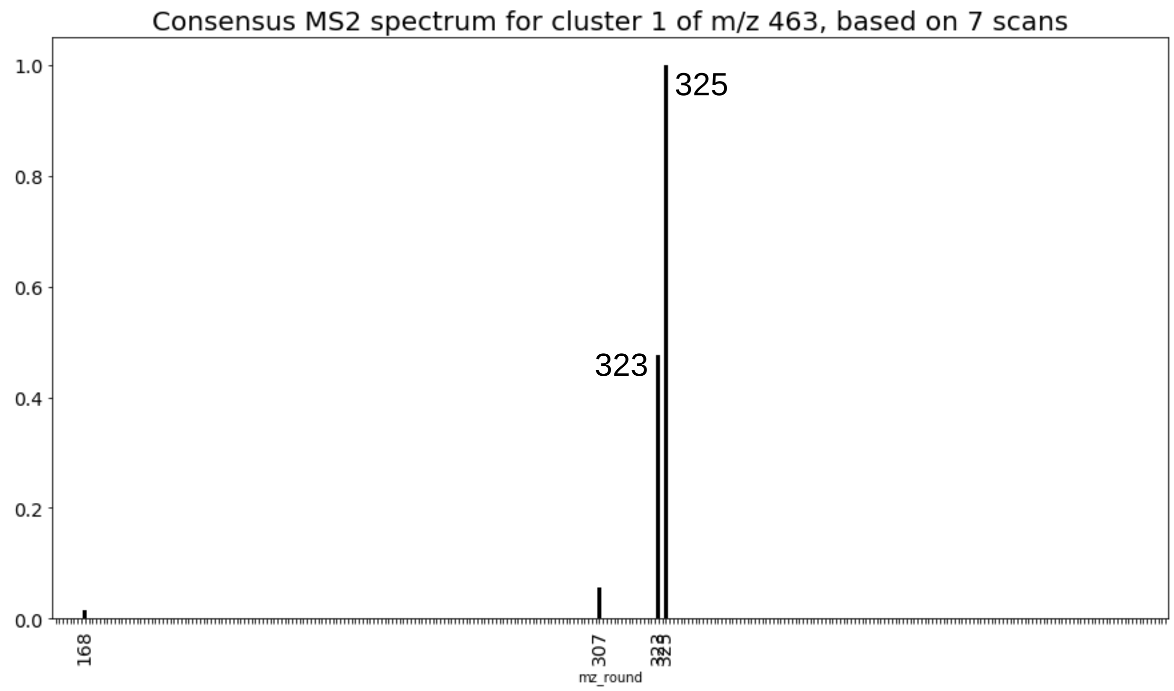

Figure S21. Doscadenamide H (**8**) consensus MS^2 spectrum
Consensus MS^2 spectrum representing a cluster of 7 scans for precursor mass *m*/*z* 463, representing the fragmentation spectrum of the proposed analog doscadenamide H (**8**).

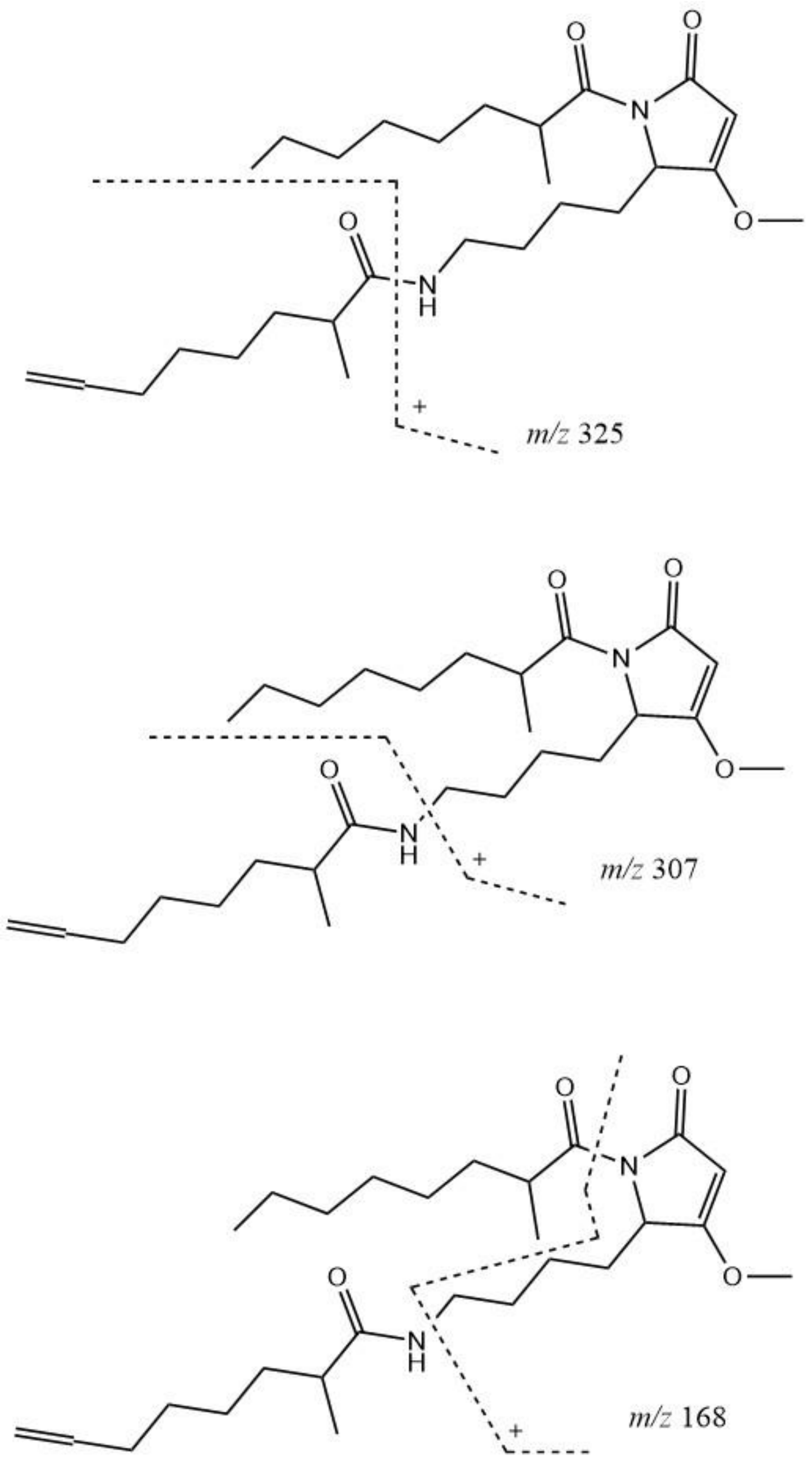

Figure S22. Doscadenamide H (**8**) proposed fragmentation

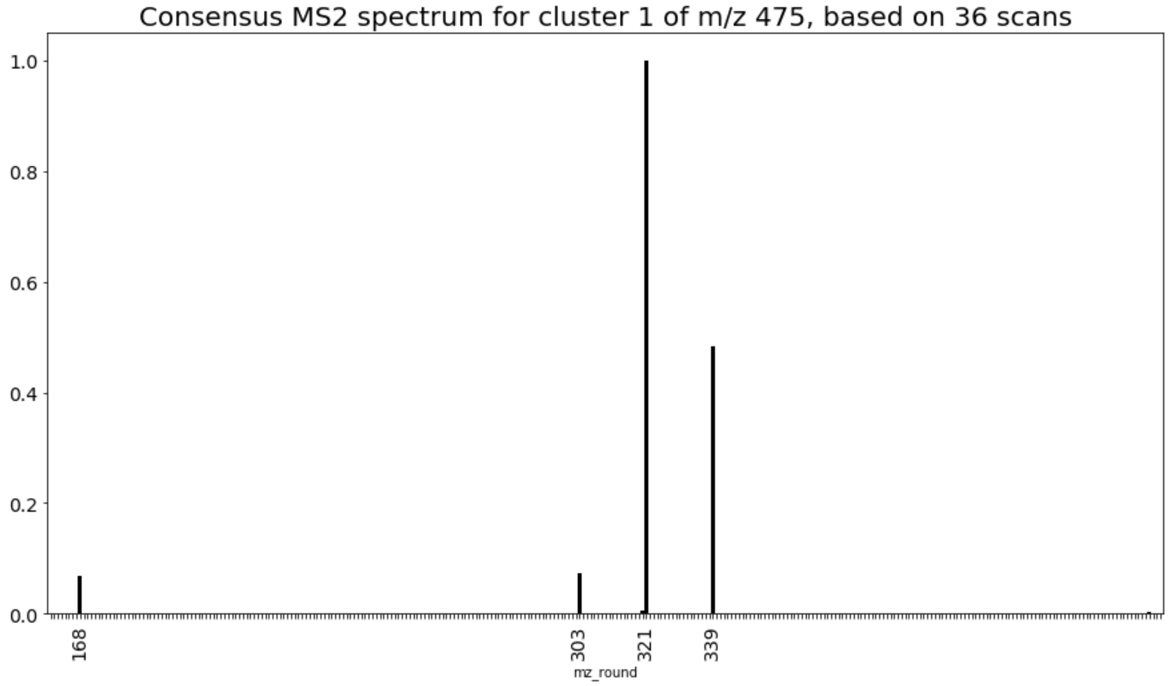

Figure S23. Doscadenamide I (**9**) consensus MS² spectrum
Consensus MS² spectrum representing a cluster of 36 scans for precursor mass *m*/*z* 475, representing the fragmentation spectrum of the proposed analog doscadenamide I (**9**).

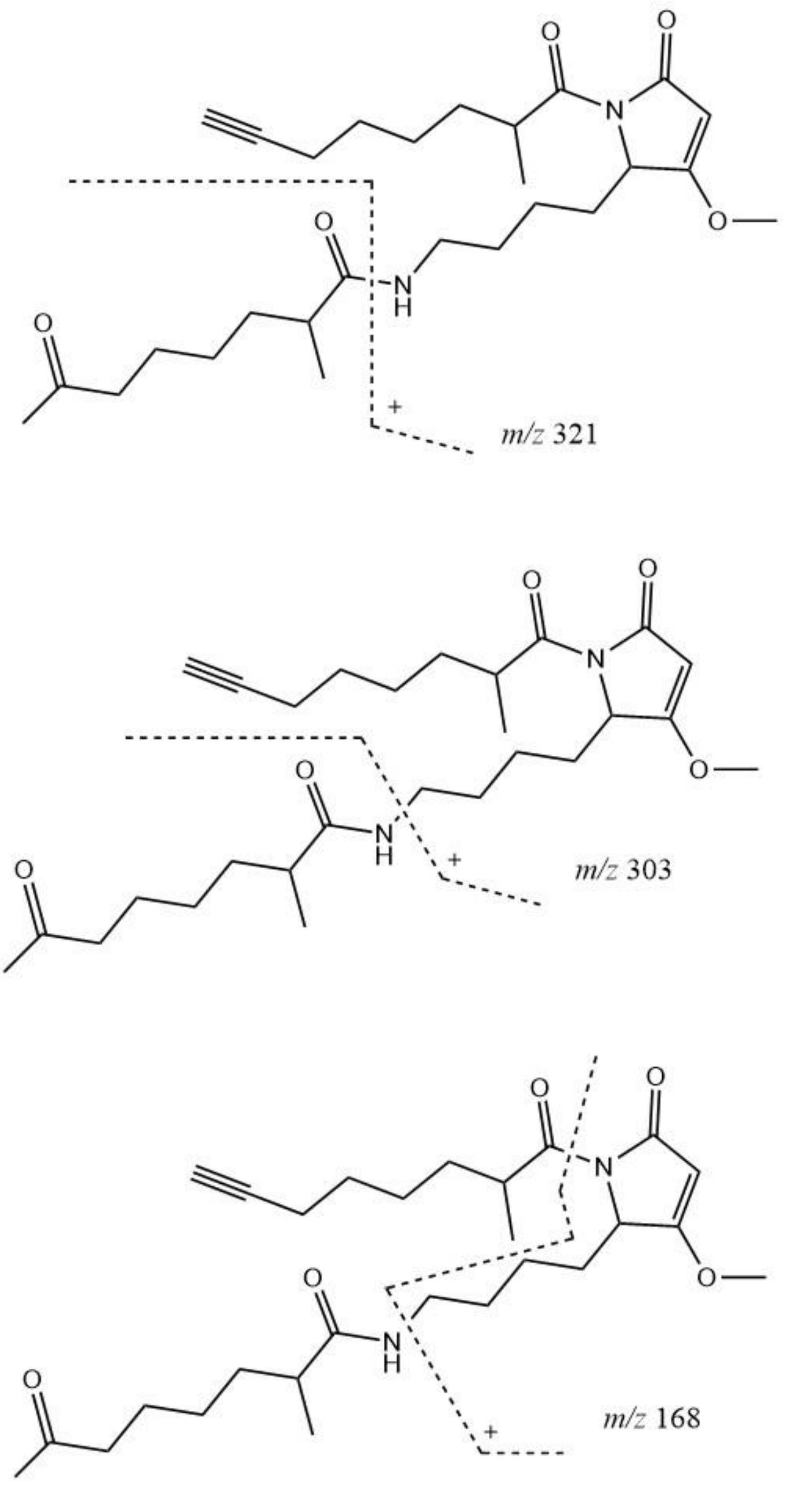

Figure S24. Doscadenamide I (**9**) proposed fragmentation

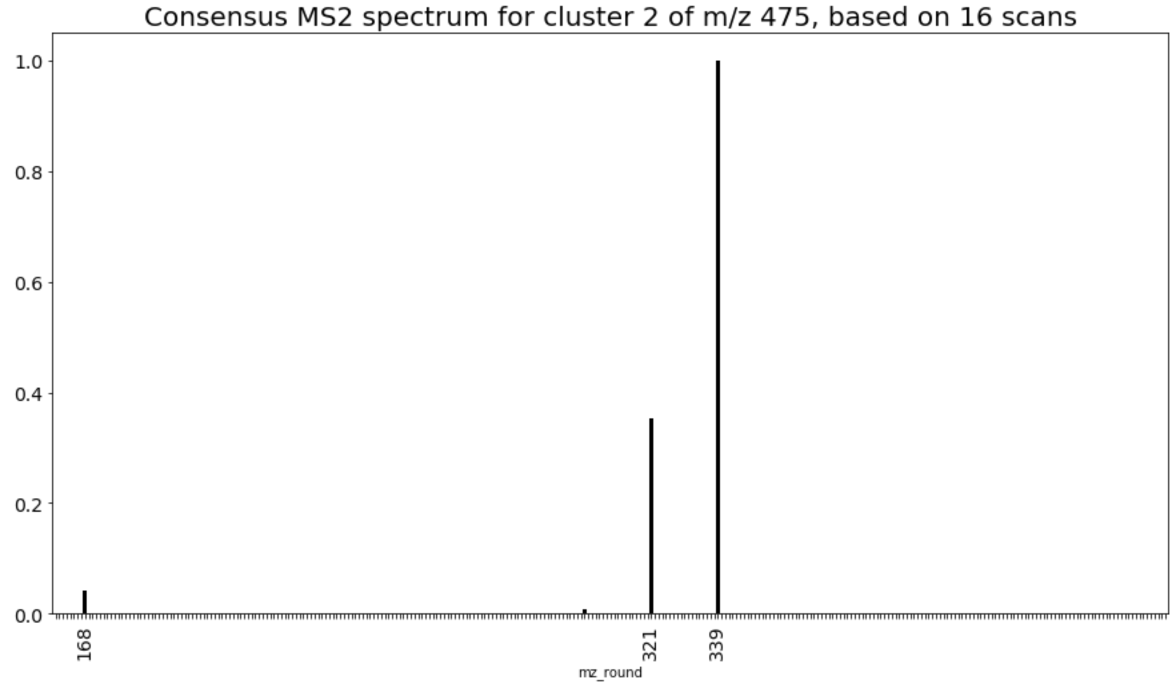

Figure S25. Doscadenamide J (**10**) consensus MS^2 spectrum
Consensus MS^2 spectrum representing a cluster of 16 scans for precursor mass *m*/*z* 475, representing the fragmentation spectrum of the proposed analog doscadenamide J (**10**).

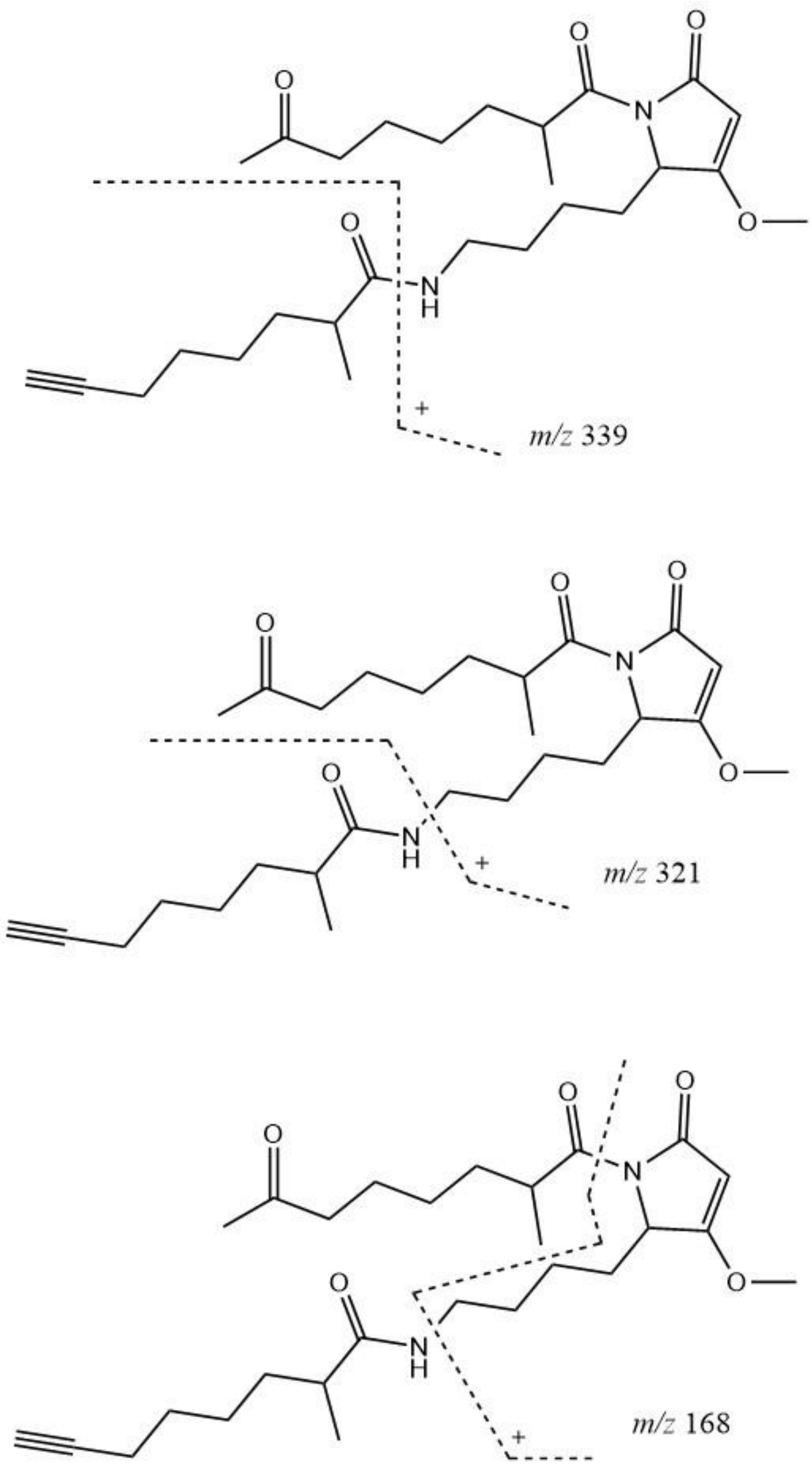

Figure S26. Doscadenamide J (**10**) proposed fragmentation

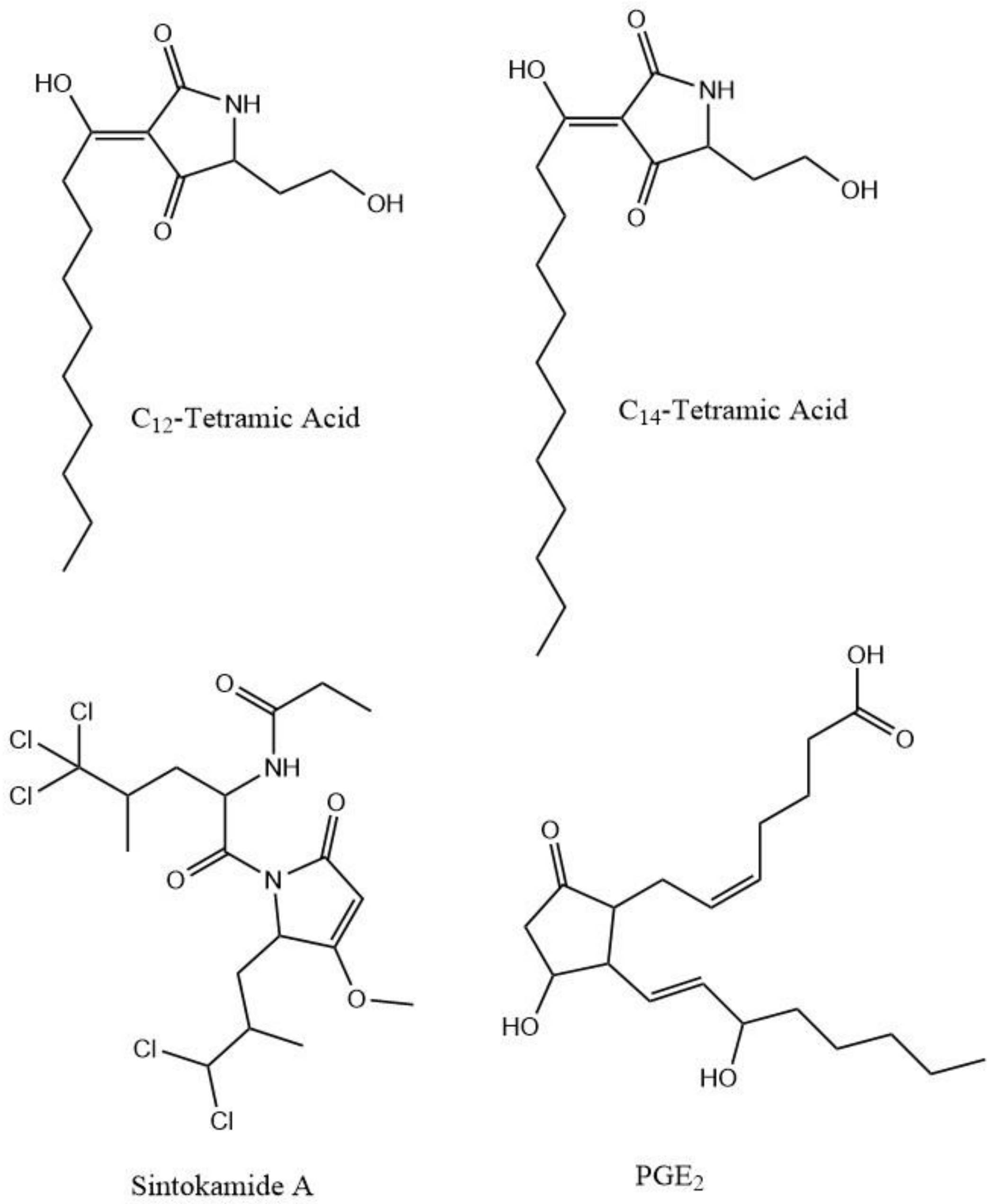

Figure S27. Representative structures from compound families similar to the doscadenamides

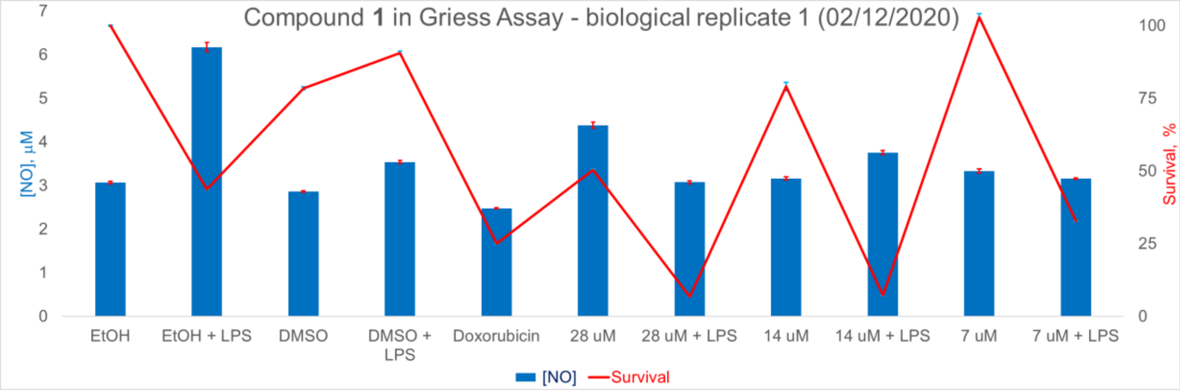

Figure S28. Results of compound **1** in Griess assay – biological replicate 1
Reagents were applied at the following concentrations: EtOH (1.5%), LPS (0.5 µg/mL), DMSO (1.0%), and doxorubicin (3.3 µg/mL). One-way ANOVA applied to the survival data indicated statistically significant differences between conditions (p-value < 0.01). Tukey's method was used to determine significance groups: EtOH (a), EtOH + LPS (b), DMSO (c), DMSO + LPS (ac), doxorubicin (d), 28 µM (b), 28 µM + LPS (e), 14 µM (c), 14 µM + LPS (e), 7 µM (a), 7 µM + LPS (d). This result indicates that when compound **1** is applied with LPS, it has a statistically significant negative impact on cell survival, as compared to compound **1** or LPS applied individually, at all three concentrations tested and in a dose-dependent fashion (e.g. when compound **1** was applied at 28 µM and 14 µM, with LPS, it had a statistically significant more negative impact on cell survival than when it was applied at 7 µM with LPS).

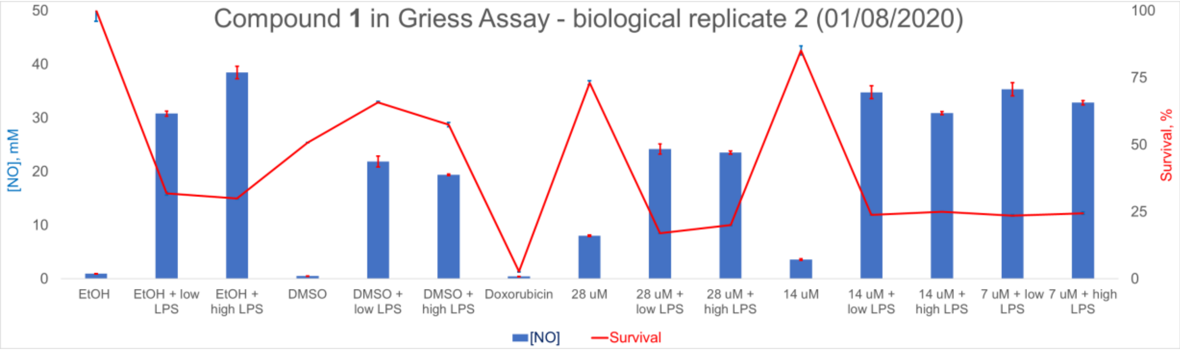

Figure S29. Results of compound **1** in Griess assay – biological replicate 2

Reagents were applied at the following concentrations: EtOH (1.0%), low LPS (0.5 µg/mL), high LPS (1.5 µg/mL), DMSO (1.0%), and doxorubicin (3.3 µg/mL). One-way ANOVA applied to the survival data indicated statistically significant differences between conditions (p-value < 0.01). Tukey's method was used to determine significance groups: EtOH (a), EtOH + low LPS (b), EtOH + high LPS (bc), DMSO (d), DMSO + low LPS (e), DMSO + high LPS (de), doxorubicin (f), 28 µM (eg), 28 µM + low LPS (c), 28 µM + high LPS (bc), 14 µM (g), 14 µM + low LPS (bc), 14 µM + high LPS (bc), 7 µM + low LPS (bc), 7 µM + high LPS (bc). No statistically significant difference was found between LPS conditions and compound **1** plus LPS conditions. However, the results still reveal a trend towards compound **1** synergistic cytotoxicity when applied with 0.5 µg/mL LPS, producing an average survival percentage of 17.1, 23.9 and 23.7% at 28, 14 and 7 µM, respectively. In comparison, 0.5 µg/mL LPS applied with EtOH resulted in an average survival of 31.8%.

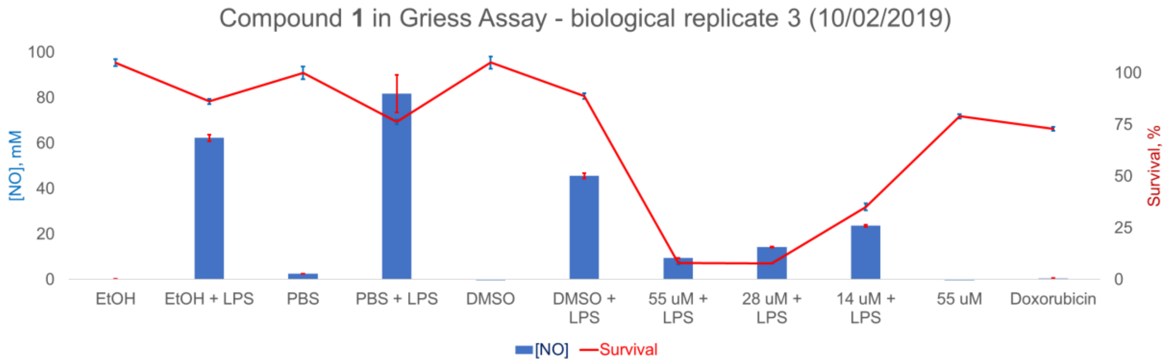

Figure S30. Results of compound **1** in Griess assay – biological replicate 3
Reagents were applied at the following concentrations: EtOH (1.0%), LPS (1.5 µg/mL), DMSO (1.0%), and doxorubicin (3.3 µg/mL). Additional control conditions were included in this assay run; phosphate-buffered saline (PBS) with and without LPS was tested. One-way ANOVA applied to the survival data indicated statistically significant differences between conditions (p-value < 0.01). Tukey's method was used to determine significance groups: EtOH (a), EtOH + LPS (ab), PBS (ab), PBS + LPS (ab), DMSO (a), DMSO + LPS (ab), 55 µM + LPS (c), 28 µM + LPS (c), 14 µM + LPS (b), 55 µM (ab), doxorubicin (b). This result illustrates the dose-dependent cytotoxicity of compound **1** when applied with LPS. Statistically significant negative impacts on cell survival were observed when compound **1** was applied with LPS at concentrations of 55 µM and 28 µM, as compared to LPS applied with negative control (EtOH or PBS) and compound **1** applied at 55 µM without LPS.

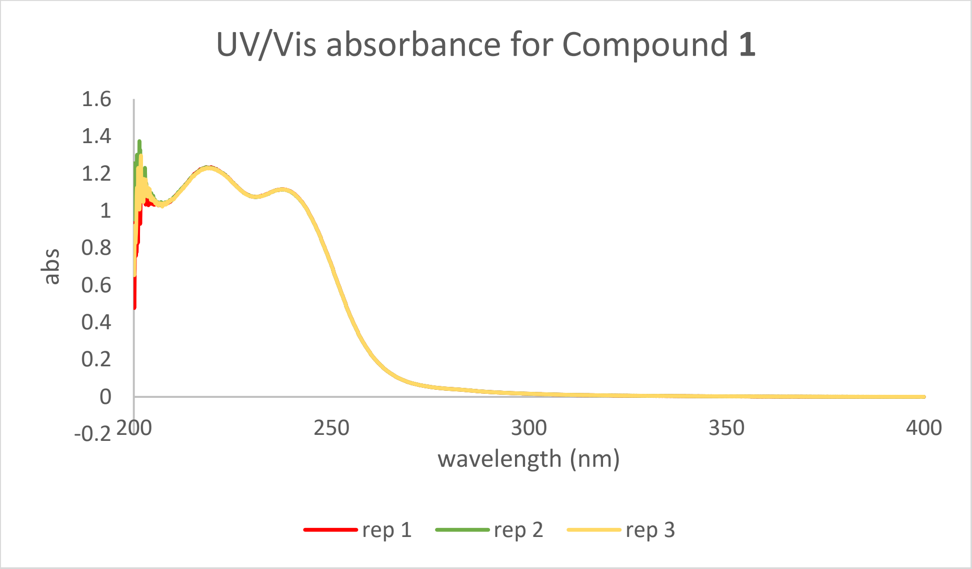

Figure S31. UV/Vis absorbance spectrum (200-400 nm) for compound **1**

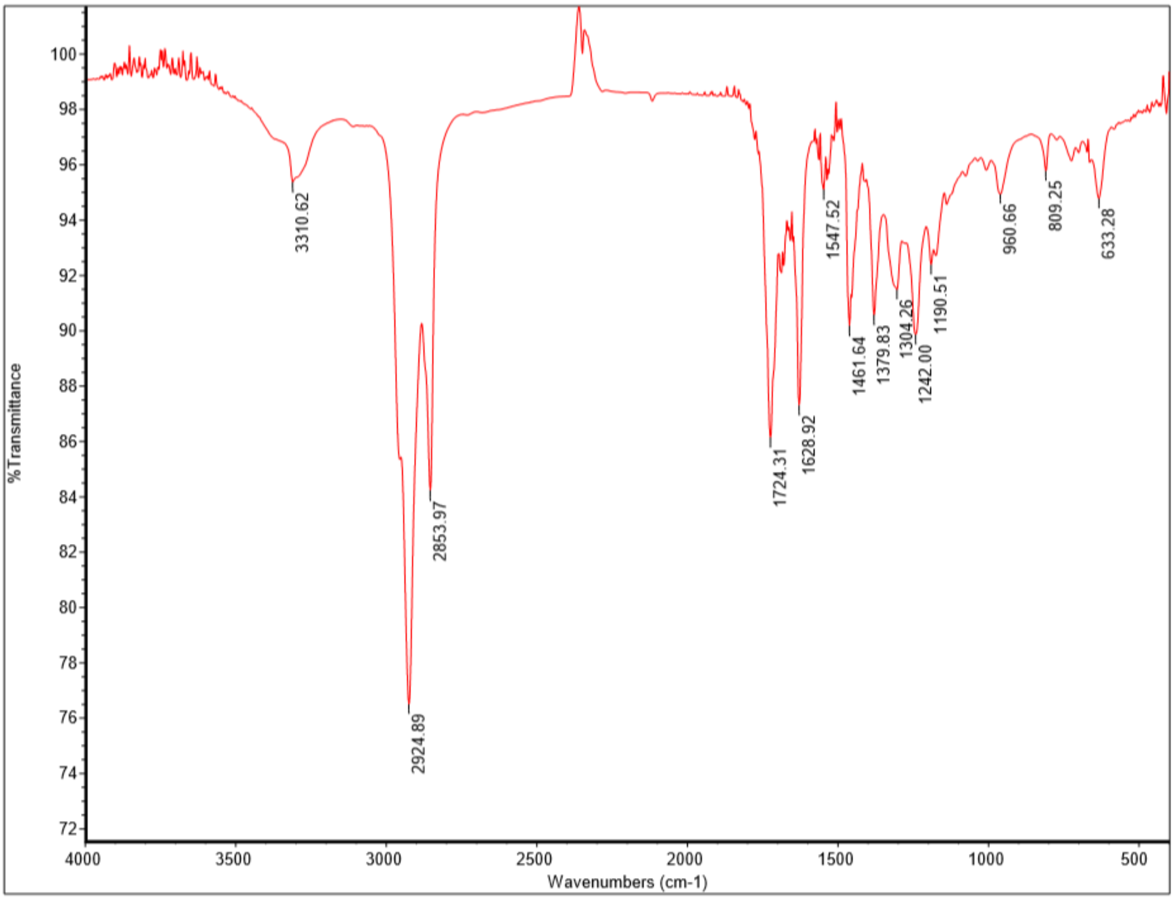

Figure S32. IR spectrum for compound **1**

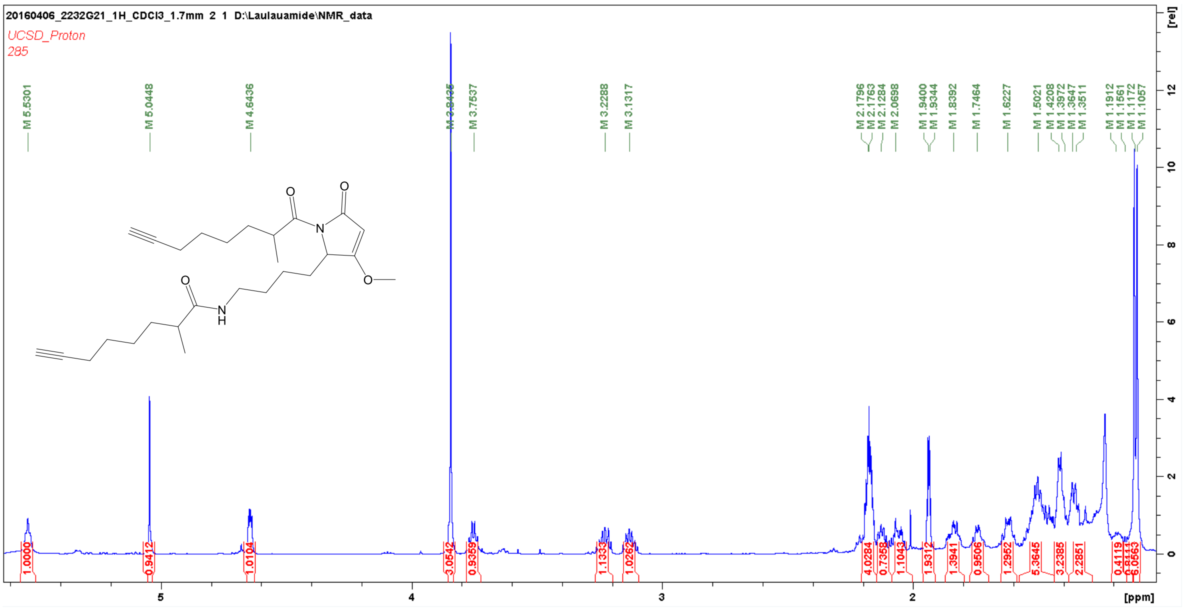

Figure S33. ¹H NMR spectrum for compound **1**

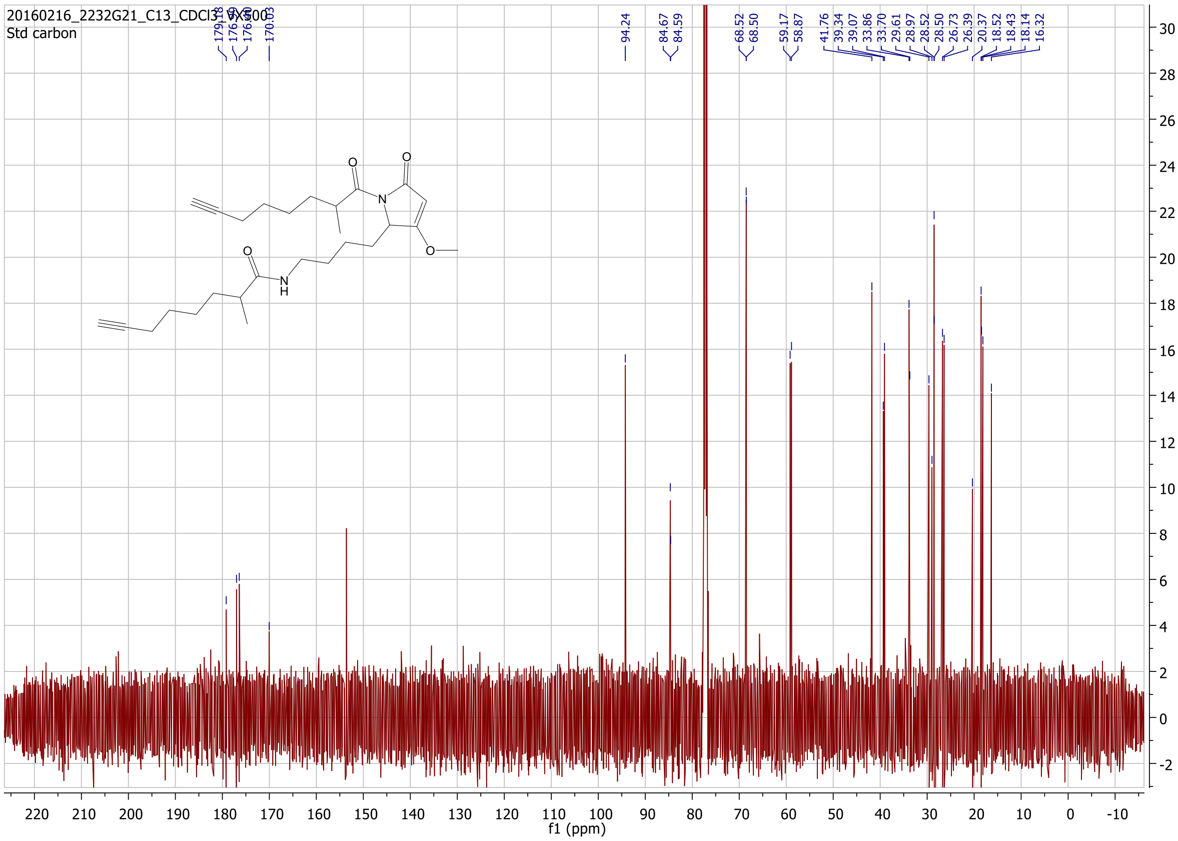

Figure S34. ^{13}C NMR spectrum for compound **1**

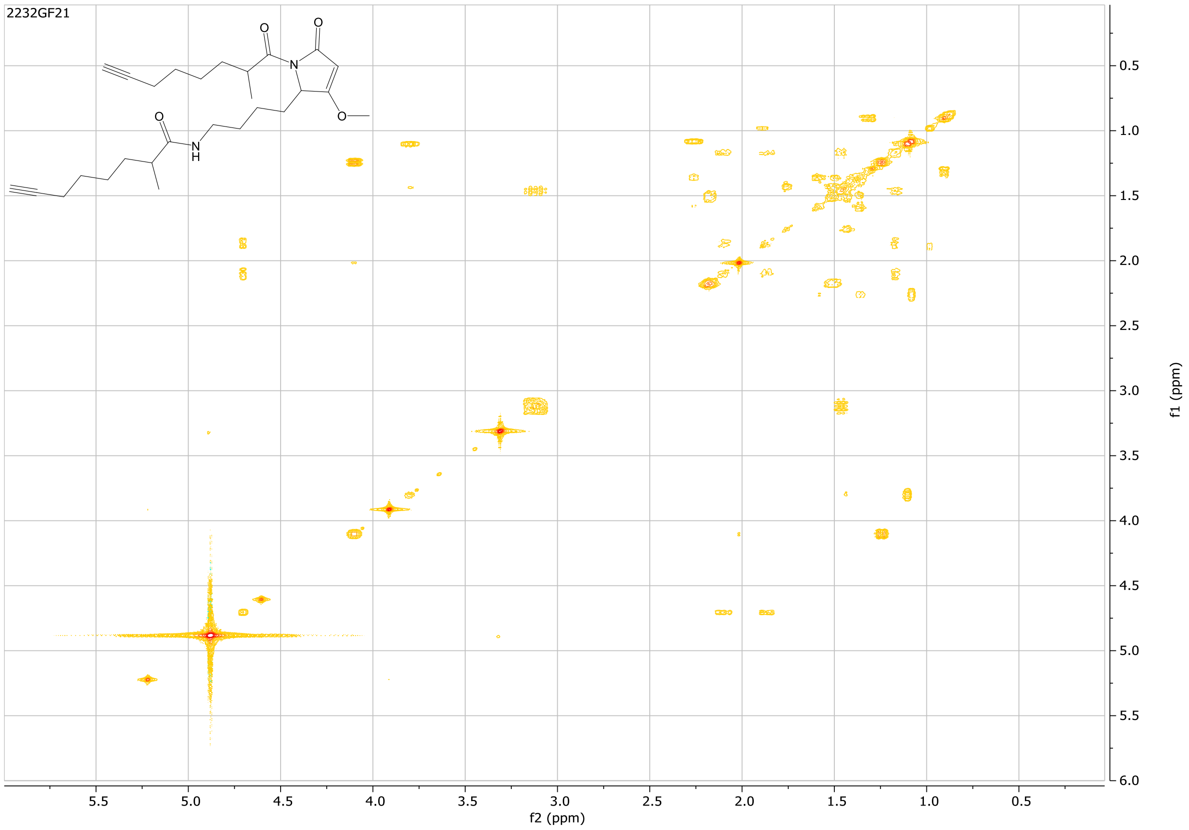

Figure S35. ^{1}H-^{1}H COSY spectrum for compound **1**

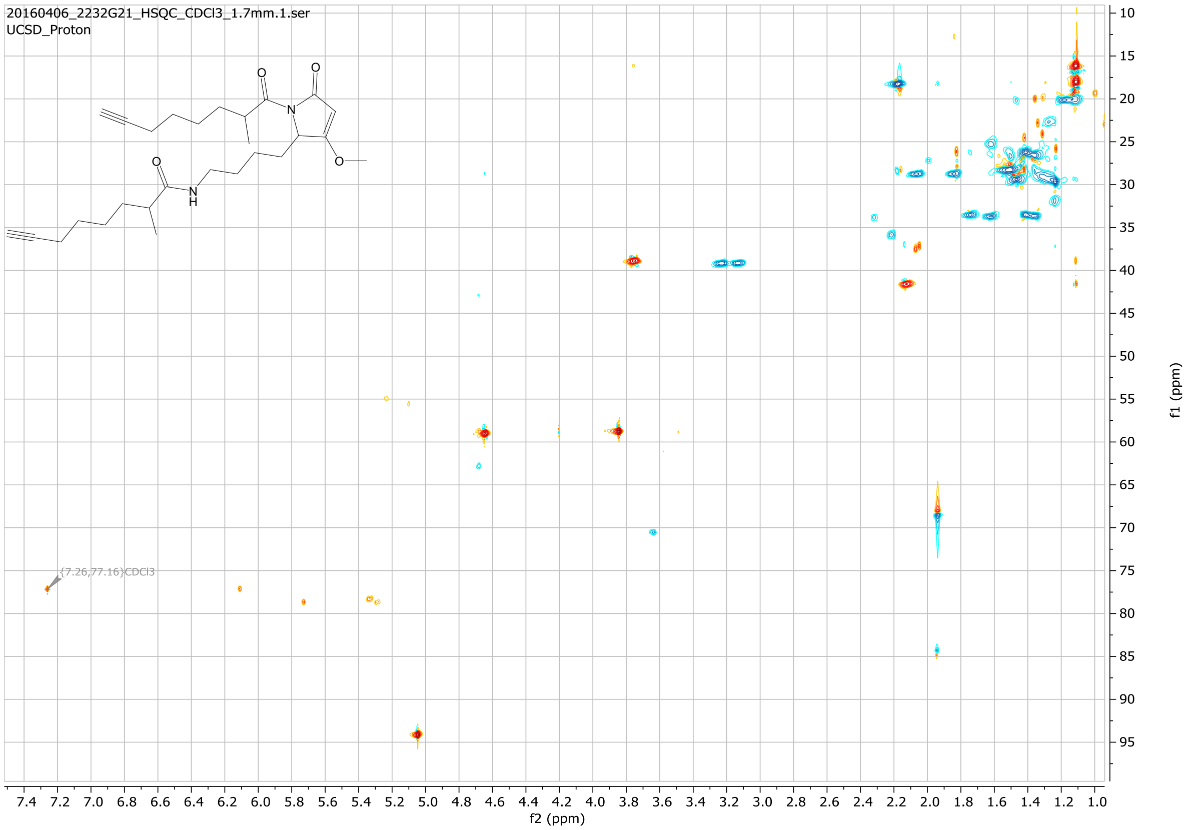

Figure S36. ^{1}H-^{13}C HSQC spectrum for compound **1**

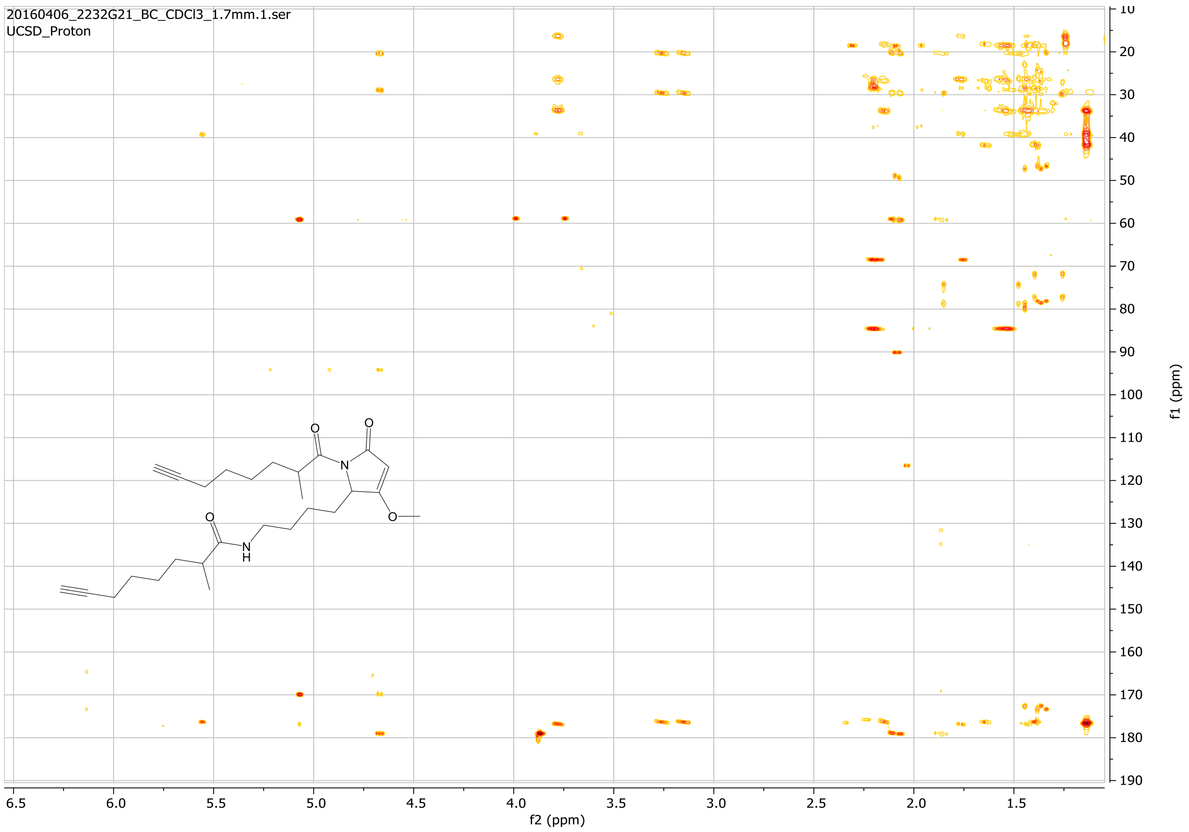

Figure S37. ^{1}H-^{13}C HMBC spectrum for compound **1**

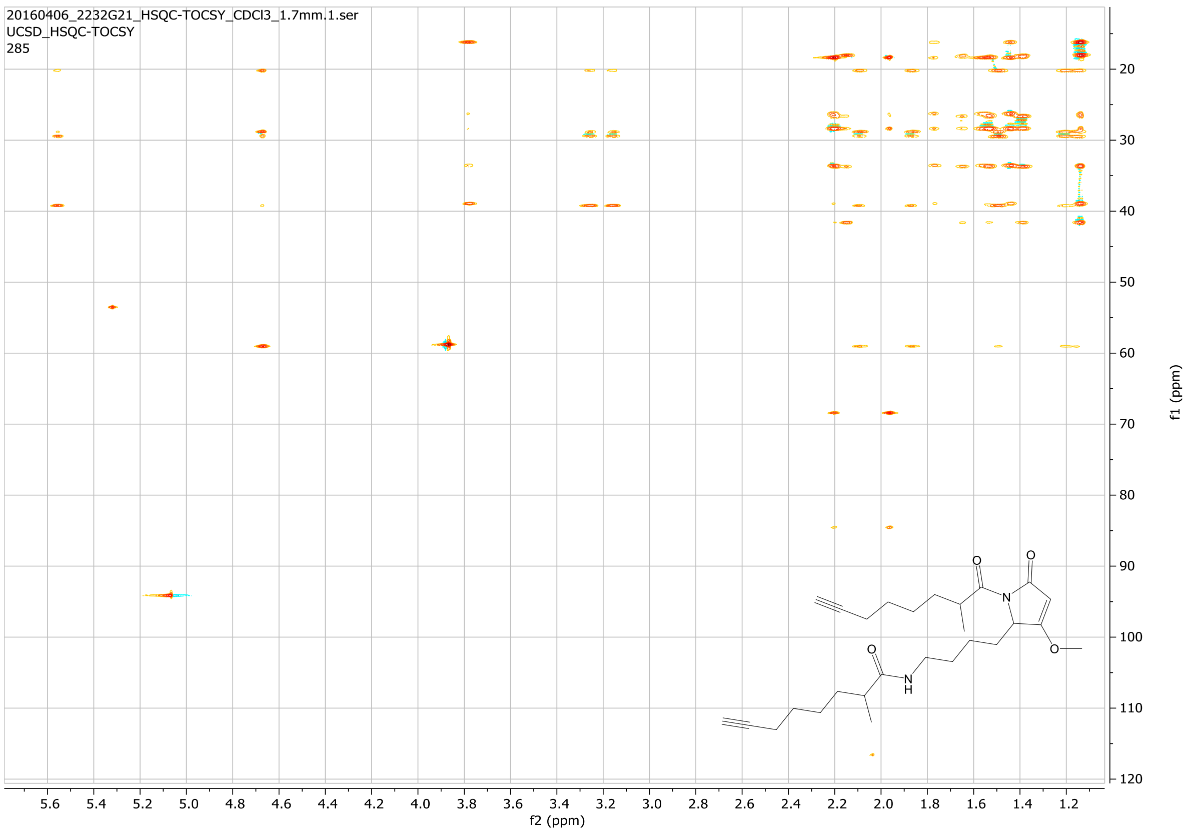

Figure S38. ^{1}H-^{13}C HSQC-TOCSY spectrum for compound **1**

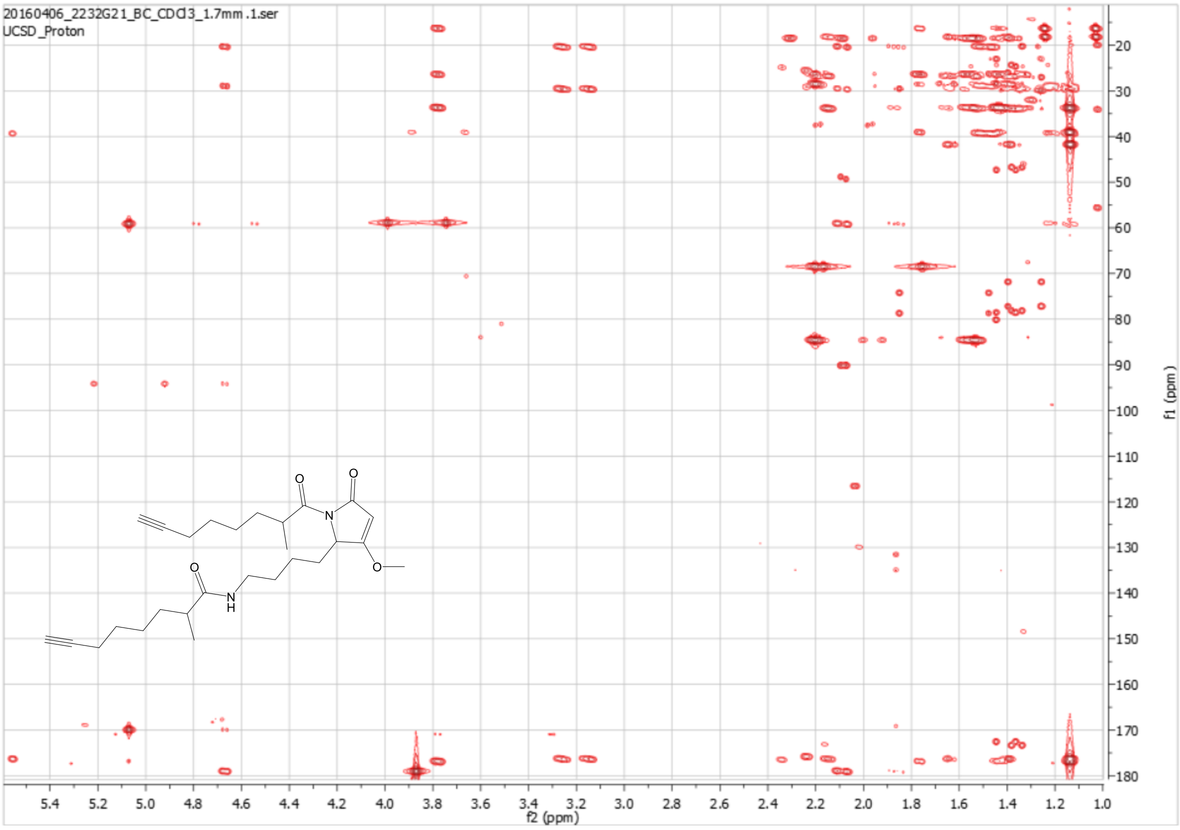

Figure S39. ^{1}H-^{13}C Long-range HSQMBC spectrum for compound **1**

Table S1. Known compounds isolated from *M. bouillonii*

Name	Monoisotopic Mass	Protenated Peak	Sodiated Peak	Location of initial isolation	Reference	Notes
**15-norlyngbyapeptin A**	683.3716	684.3786	706.3608	Palau, Guam	[11]	1
**18E-lyngbyaloside C**	648.2509	649.2579	671.2401	Guam	[12]	
**18Z-lyngbyaloside C**	648.2509	649.2579	671.2401	Guam	[12]	
**27-deoxylyngbyabellin A**	674.1766	675.1836	697.1658	Guam	[12]	
**2-epi-lyngbyaloside**	660.2509	661.2579	683.2401	Guam	[12]	
**7-epilyngbyabellin L**	544.1105	545.1175	567.0997	Palmyra Atoll	[13]	
**alotamide A**	587.3393	588.3463	610.3285	Papua New Guinea	[14]	
**apramide A**	976.5819	977.5889	999.5711	Guam	[15]	
**apramide B**	962.5663	963.5733	985.5555	Guam	[15]	
**apramide C**	978.5976	979.6046	1001.5868	Guam	[15]	
**apramide D**	1002.5976	1003.6046	1025.5868	Guam	[15]	
**apramide E**	988.5819	989.5889	1011.5711	Guam	[15]	
**apramide F**	1004.6132	1005.6202	1027.6024	Guam	[15]	
**apramide G**	827.5343	828.5413	850.5235	Guam	[15]	
**apratoxin A**	839.4866	840.4936	862.4758	Guam	[16]	2
**apratoxin A sulfoxide**	855.4816	856.4886	878.4708	Red Sea	[17]	3
**apratoxin B**	825.471	826.4780	848.4602	Guam	[18]	4
**apratoxin C**	825.471	826.4780	848.4602	Palau	[18]	4
**apratoxin D**	882.5415	883.5485	905.5307	Papua New Guinea	[19]	5
**apratoxin E**	795.4604	796.4674	818.4496	Guam	[20]	
**apratoxin F**	827.4866	828.4936	850.4758	Palmyra Atoll	[21]	
**apratoxin G**	813.471	814.4780	836.4602	Palmyra Atoll	[21]	
**apratoxin H**	853.5023	854.5093	876.4915	Red Sea	[17]	3
**apratyramide**	804.4309	805.4379	827.4201	Guam	[22]	
**bouillonamide**	817.49896	818.5060	840.4882	Papua New Guinea	[23]	
**bouillomide A (lyngbyastatin 9)**	960.4956	961.5026	983.4848	Guam	[24]	
**bouillomide B (lyngbyastatin 10)**	1038.4062	1039.4132	1061.3954	Guam	[24]	
**columbamide A**	465.2413	466.2483	488.2305	Papua New Guinea	[25]	
**columbamide B**	499.2023	500.2093	522.1915	Papua New Guinea	[25]	
**columbamide C**	423.2307	424.2377	446.2199	Papua New Guinea	[25]	
**columbamide D**	451.262	452.2690	474.2512	Malaysia	[26]	
**columbamide E**	485.223	486.2300	508.2122	Malaysia	[26]	
**columbamide F**	493.2726	494.2796	516.2618	Malaysia	[27]	
**columbamide G**	527.2336	528.2406	550.2228	Malaysia	[27]	
**columbamide H**	417.301	418.3080	440.2902	Malaysia	[27]	
**cyanolide A**	832.482	833.4890	855.4712	Papua New Guinea	[28]	
**doscadenamide A**	456.2988	457.3058	479.2880	Guam	[29]	
**kakeromamide A**	790.4088	791.4158	813.3980	Japan	[30]	
**kakeromamide B**	790.4088	791.4158	813.3980	Fiji	[31]	6
**kanamienamide**	492.3563	493.3633	515.3455	Japan	[32]	
**laingolide**	351.2773	352.2843	374.2665	Papua New Guinea	[33]	
**laingolide A**	337.2617	338.2687	360.2509	Papua New Guinea	[34]	
**laingolide B**	369.2071	370.2141	392.1963	Guam	[12]	
**lyngbouilloside**	584.356	585.3630	607.3452	Papua New Guinea	[35]	
**lyngbyabellin A**	690.1715	691.1785	713.1607	Guam	[36]	2
**lyngbyabellin B**	678.1715	679.1785	701.1607	Guam	[37]	2
**lyngbyabellin C**	608.082	609.0890	631.0712	Palau	[38]	1
**lyngbyabellin D**	895.2553	896.2623	918.2445	Palau, Guam	[11]	1
**lyngbyabellin J**	863.2291	864.2361	886.2183	Guam	[12]	
**lyngbyabellin K**	578.0715	579.0785	601.0607	Palmyra Atoll	[13]	
**lyngbyabellin L**	544.1105	545.1175	567.0997	Palmyra Atoll	[13]	
**lyngbyabellin M**	624.1133	625.1203	647.1025	Palmyra Atoll	[13]	
**lyngbyabellin N**	904.292	905.2990	927.2812	Palmyra Atoll	[13]	
**lyngbyaloside**	660.2509	661.2579	683.2401	Papua New Guinea	[39]	
**lyngbyaloside B**	648.2509	649.2579	671.2401	Palau	[40]	1
**lyngbyapeptin A**	697.3873	698.3943	720.3765	Papua New Guinea	[41]	
**lyngbyapeptin B**	721.3509	722.3579	744.3401	Palau	[38]	1
**lyngbyapeptin C**	735.3665	736.3735	758.3557	Palau	[38]	1
**lyngbyapeptin D**	683.3716	684.3786	706.3608	Guam	[12]	

**lyngbyastatin 2**	1058.6878	1059.6948	1081.6770	Guam	[42]	2
**mandangolide**	377.561	378.5680	400.5502	Papua New Guinea	[34]	
**mooreamide A**	389.293	390.3000	412.2822	Papua New Guinea	[43]	
**norlyngbyastatin 2**	1044.6722	1045.6792	1067.6614	Guam	[42]	2
**palau'imide**	428.2675	429.2745	451.2567	Palau	[38]	1
**ulongamide A**	627.309	628.3160	650.2982	Palau	[44]	1
**ulongamide B**	643.3039	644.3109	666.2931	Palau	[44]	1
**ulongamide C**	691.3039	692.3109	714.2931	Palau	[44]	1
**ulongamide D**	671.3352	672.3422	694.3244	Palau	[44]	1
**ulongamide E**	685.3509	686.3579	708.3401	Palau	[44]	1
**ulongamide F**	607.3403	608.3473	630.3295	Palau	[44]	1

[1]Reported as *Lyngbya sp.*; cited in subsequent publications as *M. bouillonii*

[2]Reported as *Lyngbya majuscula*; cited in subsequent publications as *M. bouillonii*

[3]Reported as *Moorea producens*; 16S classification inconclusive; chemistry associated with *M. bouillonii*

[4]Reported as *Lyngbya sp.*; but cited in subsequent publications as *M. bouillonii* and reported to grow with *Alpheus frontalis*

[5]Reported as *Lyngbya majuscula* and *Lyngbya sordid*; 16S classification inconclusive; chemistry associated with *M. bouillonii*

[6]Reported as *Moorea producens*; manuscript includes a photo of woven *M. bouillonii*; 16S classification inconclusive; compound isolated along with known compounds previously isolated from *M. bouillonii*

Table S2. Average relative abundances and feature selection scores for top 10 Saipan MS^1 features

m/z	relative rt	F-value[1]	p-value[1]	American Samoa[2]	China[2]	Guam[2]	India[2]	PNG[2]	Saipan[2]
721.10	0.6065	0.869	0.5371	0.045	0.025	0	0	0.007	0.210
**457.05**[3]	**0.4852**	**17.402**	**0.0002**	**0.012**	**0.023**	**0.006**	**0**	**0.005**	**0.176**
1368.03	0.5654	5.049	0.0176	0.011	0.035	0.014	0.008	0.005	0.175
609.10	0.6263	1.229	0.3704	0.049	0.028	0.050	0.126	0.010	0.172
535.10	0.6177	2.073	0.1613	0.065	0.034	0.214	0.005	0	0.158
1367.06	0.5626	2.314	0.1296	0.008	0.027	0.030	0.069	0	0.136
459.06	0.5141	31.678	< 0.0001	0	0.024	0.006	0.005	0	0.112
536.10	0.6270	3.205	0.0617	0.094	0.014	0.034	0.006	0.006	0.102
1687.01	0.4942	1.086	0.4296	0.005	0.008	0	0.019	0.005	0.100
722.04	0.5938	0.703	0.6356	0.035	0.010	0.015	0.008	0.004	0.096

[1]F-values and p-values are generated in ORCA using the scikit learn (https://scikit-learn.org/stable/) implementation of univariate feature selection. These scores should be interpreted cautiously, as the dataset does not meet the assumptions necessary for univariate features selection, but can still help in generating hypotheses about which features are driving differences between samples collected from different geographical regions.

[2]Average of the unit vector normalized integrated feature values for all samples from the geographical region.

[3]Compound **1** was detected in high abundance in samples from Saipan, ranking as the second most abundant MS^1 feature, while not being detected or being detected at very low levels in other samples.

Table S3. Putative identifications for the top 30 MS^1 features in the *M. bouillonii* crude extract dataset

	m/z	relative rt	max transformed integral	putative ids	difference
1	815.10	0.6383	0.857928	['apratoxin G [M+H]+']	[0.62]
2	814.06	0.5968	0.765564	['apratoxin G [M+H]+', 'kakeromamide A [M+Na]+', 'kakeromamide B [M+Na]+']	[0.42, 0.66, 0.66]
3	721.10	0.6065	0.572637	['lyngbyapeptin A [M+Na]+']	[0.73]
4	840.07	0.6350	0.510516	['apratoxin A [M+H]+', 'bouilllonamide [M+Na]+']	[0.43, 0.42]
5	862.08	0.5894	0.508103	['apratoxin A [M+Na]+']	[0.39]
6	623.10	0.6501	0.440651	['None']	[0]
7	611.09	0.5845	0.427792	['alotamide A [M+Na]+']	[0.77]
8	678.10	0.5988	0.386879	['None']	[0]
9	535.10	0.6177	0.382478	['None']	[0]
10	609.10	0.6263	0.369167	['lyngbyabellin C [M+H]+', 'ulongamide F [M+H]+']	[0.01, 0.75]
11	378.06	0.5832	0.35347	['mandangolide [M+H]+']	[0.5]
12	625.11	0.6320	0.334295	['lyngbyabellin M [M+H]+']	[0.01]
13	793.11	0.6292	0.326725	['None']	[0]
14	827.10	0.6185	0.312621	['apratoxin B [M+H]+', 'apratoxin C [M+H]+', 'apratyramide [M+Na]+']	[0.62, 0.62, 0.32]
15	744.09	0.6160	0.31144	['lyngbyapeptin B [M+Na]+']	[0.25]
16	836.08	0.6534	0.286632	['apratoxin G [M+Na]+']	[0.38]
17	1368.02	0.5654	0.280465	['None']	[0]
18	639.08	0.6220	0.279184	['None']	[0]
19	581.10	0.6134	0.277933	['None']	[0]
20	632.10	0.6188	0.274745	['None']	[0]
21	722.04	0.5938	0.266215	['lyngbyapeptin B [M+H]+']	[0.32]
22	886.05	0.6135	0.258829	['lyngbyabellin J [M+Na]+']	[0.17]
23	841.13	0.6118	0.24873	['apratoxin A [M+H]+', 'bouilllonamide [M+Na]+']	[0.63, 0.64]
24	651.11	0.6804	0.246204	['ulongamide A [M+Na]+']	[0.81]
25	1687.01	0.4942	0.245016	['None']	[0]
**26**	**457.05**	**0.4852**	**0.242068**	**['None']**[1]	**[0]**
27	1367.06	0.5627	0.22625	['None']	[0]
28	816.09	0.6681	0.224017	['None']	[0]
29	494.07	0.6387	0.21453	['columbamide F [M+H]+', 'kanamienamide [M+H]+']	[0.21, 0.71]
30	863.09	0.6430	0.209889	['apratoxin A [M+Na]+']	[0.61]

[1]No putative identifications were assigned to compound **1**, suggesting it to be a new natural product.

Table S4. *In silico* antibiotic screening results for the doscadenamides and tetramic acids

compound name	SMILES	score[1]
doscadenamides A	COC1=CC(N(C(C(C(C)CCCCC#C)=O)C1CCCCNC(C(C)CCCCC#C)=O)=O	0.031352468
doscadenamides B	COC1=CC(N(C(C(C(C)CCCCC=C)=O)C1CCCCNC(C(C)CCCCC=C)=O)=O	0.056482284
doscadenamides C	COC1=CC(N(C(C(C(C)CCCCC#C)=O)C1CCCCNC(C(C)CCCCC=C)=O)=O	0.038773874
doscadenamides D	COC1=CC(N(C(C(C(C)CCCCC=C)=O)C1CCCCNC(C(C)CCCCC#C)=O)=O	0.038728081
doscadenamides E	COC1=CC(N(C(C(C(C)CCCCC#C)=O)C1CCCCNC(C(C)CCCCCC)=O)=O	0.062902918
doscadenamides F	COC1=CC(N(C(C(C(C)CCCCCC)=O)C1CCCCNC(C(C)CCCCC#C)=O)=O	0.0636456
doscadenamides G	COC1=CC(N(C(C(C(C)CCCCC=C)=O)C1CCCCNC(C(C)CCCCCC)=O)=O	0.091586996
doscadenamides H	COC1=CC(N(C(C(C(C)CCCCCC)=O)C1CCCCNC(C(C)CCCCC=C)=O)=O	0.092198204
doscadenamides I	COC1=CC(N(C(C(C(C)CCCCC#C)=O)C1CCCCNC(C(C)CCCCC(C)=O)=O)=O	0.049369105
doscadenamides J	COC1=CC(N(C(C(C(C)CCCCC(C)=O)=O)C1CCCCNC(C(C)CCCCC#C)=O)=O	0.04936365
C_{12}-tetramic acid	O=C(/C1=C(O)/CCCCCCCCC)NC(CCO)C1=O	0.545725947
C_{14}-tetramic acid	O=C(/C1=C(O)/CCCCCCCCCCC)NC(CCO)C1=O	0.590775286

[1] Scores represent the probability that the screened compound would inhibit *E. coli* growth at 50 μM [78].

Table S5. *M. bouillonii* crude extract sample metadata

filename	alias	extract number	collection code	collection country/territory	collection region	collection site	collection date
20170915_CBN_XSSCB2017_13.mzXML	China_13	-	XSSCB2017_13	China/Xisha	Sanshax	16° 51' 05.52", 112° 20' 56.13"	5/16/2017
20170915_CBN_XSSCB2017_24.mzXML	China_24	-	XSSCB2017_24	China/Xisha	Sanshax	16° 51' 05.52", 112° 20' 56.13"	5/19/2017
20170915_CBN_XSSCB2017_25.mzXML	China_25	-	XSSCB2017_25	China/Xisha	Sanshax	16° 51' 05.52", 112° 20' 56.13"	5/19/2017
2019-08-23_CBN_KHI-18-1.mzXML	India_KHI	-	KHT08APR18-3	India/Lakshadweep	Kavaratti	Heaven's Treat lagoon	4/8/2018
2019-08-23_CBN_KP-16-1.mzXML	India_KP	-	KP-16	India/Lakshadweep	Kavaratti	Paradise Hut lagoon	2/6/2016
2019-08-23_CBN_KPL-18-1.mzXML	India_KPL	-	KPL08APR18-1	India/Lakshadweep	Kavaratti	Paradise Hut lagoon	4/8/2018
2019-08-23_CBN_KSP-18-1.mzXML	India_KSP	-	KSP07APR18-1	India/Lakshadweep	Kavaratti	south of Paradise Hut pier	4/7/2018
2200.mzXML	Saipan_00	2200	SPB31JAN13-1	Saipan	-	Laulau Bay	1/31/2013
2209.mzXML	Saipan_09	2209	SPD29JAN13-6	Saipan	-	Laulau Bay	1/29/2013
2220.mzXML	AmSam_20	2220	ASA12JUL14-1	American Samoa	-	Afao	7/12/2014
2223.mzXML	AmSam_23	2223	ASG15JUL14-1	American Samoa	-	Fagasa Bay	7/15/2014
2232.mzXML	Saipan_32	2232	SPB01FEB13-1	Saipan	-	Laulau Bay	2/1/2013
2246.mzXML	Guam_46	2246	GBB21MAR16-1	Guam	-	Apra Harbor	3/21/2016
2247.mzXML	Guam_47	2247	GGG21MAR16-1	Guam	-	Apra Harbor	3/21/2016
Mb.mzXML	PNG_c	-	PNG19MAY05-8	Papua New Guinea	New Ireland	Pigeon Island	5/19/2005

Table S6. ORCA parameter set for MS^1 feature dendrogram

parameter	value
bin_width	0.5
bin_offset	0
bins_start	200
bins_end	2000
peak_consecutivity	0
peak_cluster_size_cutoff	3
min_integral	100000
rt_setting	‘relative’
rrt_tolerance	0.05
transforms	None
metric	‘cosine’
method	‘average’
color_cutoff	N/A (custom colorization)

Table S7. ORCA parameter set for GNPS MS^2 feature presence/absence dendrogram

parameter	value
drop_columns	['#OTU ID']
drop_rows	[]
transpose_buckettable	False
transforms	presence_absence = True
metric	'cosine'
method	'average'
color_cutoff	N/A (custom colorization)

Table S8. ORCA parameter set for MS^1 feature selection

parameter	value
bin_width	1
bin_offset	0
bins_start	200
bins_end	2000
peak_consecutivity	0
peak_cluster_size_cutoff	3
min_integral	100000
rt_setting	'relative'
rrt_tolerance	0.05
transforms	None
metric	'cosine'
method	'average'
color_cutoff	N/A (custom colorization)

Table S9. GNPS parameter set for *M. bouillonii* crude extract molecular network

parameter	value
workflow version	1.2.5
PAIRS_MIN_COSINE	0.7
ANALOG_SEARCH	1
tolerance.PM_tolerance	2.0
tolerance.Ion_tolerance	0.5
MIN_MATCHED_PEAKS	2
TOPK	10
CLUSTER_MIN_SIZE	1
MAXIMUM_COMPONENT_SIZE	100
MIN_PEAK_INT	50
FILTER_STDDEV_PEAK_INT	0.0
RUN_MSCLUSTER	on
FILTER_PRECURSOR_WINDOW	1
FILTER_LIBRARY	1
WINDOW_FILTER	1
SCORE_THRESHOLD	0.7
MIN_MATCHED_PEAKS_SEARCH	2
MAX_SHIFT_MASS	100.0

Table S10. GNPS parameter set for *M. bouillonii* crude extract MS^2 feature bucket table

parameter	value
workflow version	release_22
PAIRS_MIN_COSINE	0.7
ANALOG_SEARCH	1
tolerance.PM_tolerance	2.0
tolerance.Ion_tolerance	0.5
MIN_MATCHED_PEAKS	4
TOPK	10
CLUSTER_MIN_SIZE	1
MAXIMUM_COMPONENT_SIZE	100
MIN_PEAK_INT	0.0
FILTER_STDDEV_PEAK_INT	0.0
RUN_MSCLUSTER	on
FILTER_PRECURSOR_WINDOW	1
FILTER_LIBRARY	1
WINDOW_FILTER	1
SCORE_THRESHOLD	0.7
MIN_MATCHED_PEAKS_SEARCH	4
MAX_SHIFT_MASS	100.0

Table S11. GNPS parameter set for Saipan and Guam sample crudes and fractions molecular network

parameter	value
workflow version	release_17
PAIRS_MIN_COSINE	0.7
ANALOG_SEARCH	1
tolerance.PM_tolerance	1.0
tolerance.Ion_tolerance	0.5
MIN_MATCHED_PEAKS	4
TOPK	10
CLUSTER_MIN_SIZE	2
MAXIMUM_COMPONENT_SIZE	100
MIN_PEAK_INT	0.0
FILTER_STDDEV_PEAK_INT	0.0
RUN_MSCLUSTER	on
FILTER_PRECURSOR_WINDOW	1
FILTER_LIBRARY	1
WINDOW_FILTER	1
SCORE_THRESHOLD	0.7
MIN_MATCHED_PEAKS_SEARCH	4
MAX_SHIFT_MASS	100.0

Table S12. VLC fractionation solvent systems

fraction	composition
A	100% hexane
B	90% hexane : 10% ethyl acetate
C	80% hexane : 20% ethyl acetate
D	60% hexane : 40% ethyl acetate
E	40% hexane : 60% ethyl acetate
F	20% hexane : 80% ethyl acetate
G	100% ethyl acetate
H	75% ethyl acetate : 25% methanol
I	100% methanol

Table S13. ^{1}H and ^{13}C NMR Data for doscadenamide A (**1**) in CDCl3[a]

Residue	Position	δ_C, type	δ_H, mult. (*J*, Hz)
pyLys-OMe	1	170.0, C	
	2	94.2, CH	5.04, s
	3	179.2, C	
	4	59.2, CH	4.64, dd (*J* = 5.7, 2.9 Hz)
	5	29.0, CH_2	2.07, m<br>1.84, m
	6	20.4, CH_2	1.19, m<br>1.16, m
	7	29.6, CH_2	1.44, m
	8	39.3, CH_2	3.22, dp (*J* = 19.2, 6.4 Hz)<br>3.13, dp (*J* = 19.2, 6.4 Hz)
	9	58.9, CH_3	3.84, s
	NH		5.53, brs
Moya-1	10	177.0, C	
	11	41.8, CH	2.13, m
	12	33.9, CH_2	1.62, m<br>1.35, m
	13	28.5, CH_2	1.36, m
	14	28.5, CH_2	1.50, m
	15	18.5[b], CH_2	2.18, m
	16	84.7, C	
	17	68.52[c], CH	1.934[b], t (*J* = 2.7 Hz)
	18	18.1[d], CH_3	1.11, d (*J* = 1.1 Hz)
Moya-2	19	176.4, C	
	20	39.1, CH	3.75, m
	21	33.7, CH_2	1.75, m<br>1.42, m
	22	26.7, CH_2	1.39, m
	23	26.4, CH_2	1.44, m
	24	18.4[b], CH_2	2.18, m
	25	84.6, C	
	26	68.50[c], CH	1.940[b], t (*J* = 2.7 Hz)
	27	16.3[d], CH_3	1.12, d (*J* = 1.1 Hz)

[a] Data recorded at 600 MHz (^{1}H NMR) and 125 MHz (^{13}C NMR). [b,c,d] Assignments with the same superscripted letter could be reversed.

© 2020 by the authors. Submitted for possible open access publication under the terms and conditions of the Creative Commons Attribution (CC BY) license (http://creativecommons.org/licenses/by/4.0/).
